# “Females Are Not Just ‘Protected’ Males”: Sex-Specific Vulnerabilities in Placenta and Brain after Prenatal Immune Disruption

**DOI:** 10.1523/ENEURO.0358-19.2019

**Published:** 2019-11-06

**Authors:** Amy E. Braun, Pamela A. Carpentier, Brooke A. Babineau, Aditi R. Narayan, Michelle L. Kielhold, Hyang Mi Moon, Archana Shankar, Jennifer Su, Vidya Saravanapandian, Ursula Haditsch, Theo D. Palmer

**Affiliations:** 1 Institute for Stem Cell Biology and Regenerative Medicine and Department of Neurosurgery, Stanford University, Stanford, California 94305-5454; 2 Driskill Graduate Program in Life Sciences, Feinberg School of Medicine, Northwestern University, Chicago, Illinois 60611; 3 Unity Biotechnology, Brisbane, California 94005; 4 Department of Behavioral Neuroscience, Oregon Health & Science University, Portland, Oregon 97239; 5 Department of Biological Science, California State University, Fullerton, Fullerton, California 92831; 6 Department of Cellular and Molecular Medicine, University of California, San Diego, La Jolla, California 92093; 7 Cortexyme, Inc, South San Francisco, California 94080

**Keywords:** corticogenesis, developmental origins of health and disease, female resilience, maternal immune activation, pregnancy complications, sex differences

## Abstract

Current perceptions of genetic and environmental vulnerabilities in the developing fetus are biased toward male outcomes. An argument is made that males are more vulnerable to gestational complications and neurodevelopmental disorders, the implication being that an understanding of disrupted development in males is sufficient to understand causal mechanisms that are assumed to be similar but attenuated in females. Here we examine this assumption in the context of immune-driven alterations in fetal brain development and related outcomes in female and male mice. Pregnant C57BL/6 mice were treated with low-dose lipopolysaccharide at embryonic day 12.5. Placental pathology, acute fetal brain inflammation and hypoxia, long-term changes in adult cortex cytoarchitecture, altered densities and ratio of excitatory (Satb2^+^) to inhibitory (parvalbumin^+^) neuronal subtypes, postnatal growth, and behavior outcomes were compared between male and female offspring. We find that while males experience more pronounced placental pathology, fetal brain hypoxia, depleted PV and Satb2^+^ densities, and social and learning-related behavioral abnormalities, females exhibit unique acute inflammatory signaling in fetal brain, postnatal growth delay, opposite alterations in cortical PV densities, changes in juvenile behavior, delayed postnatal body growth, and elevated anxiety-related behavior as adults. While males are more severely impacted by prenatal immune disruption by several measures, females exposed to the same insult exhibit a unique set of vulnerabilities and developmental consequences that is not present in males. Our results clearly outline disparate sex-specific features of prenatal vulnerability to inflammatory insults and warn against the casual extrapolation of male disease mechanisms to females.

## Significance Statement

Given the common practice of excluding female animals from studies of maternal immune activation during pregnancy, it appears to be widely assumed that female outcomes are simply attenuated versions of more severe male outcomes. However, when female fetuses and offspring are closely examined in a model of lipopolysaccharide-induced maternal inflammation during pregnancy, we find that sex confers selective vulnerabilities and outcomes that impact the placenta, fetal brain, adult brain, and behavior in ways that are categorically distinct and in some cases opposite between females and males. Therefore, the effect of maternal immune activation on female offspring cannot be inferred from male outcomes and must be studied independently to fully understand the mechanisms that underlie prenatal vulnerability to maternal insults.

## Introduction

In autism spectrum disorders (ASDs), the most common diagnosis in children, the current understanding is that hundreds of common alleles account for up to 60% of diagnoses, while the remaining cases are caused by “other” mechanisms (Hallmayer et al., 2011; State and Levitt, 2011; Sandin et al., 2014; Grove et al., 2019). Maladaptive immune activation events such as infection, asthma, and allergy during pregnancy have frequently been associated with poor postnatal neurodevelopmental outcomes, including autism and schizophrenia (Brown et al., 2000; Beversdorf et al., 2005; Croen et al., 2005; Atladóttir et al., 2010; Goines et al., 2011; Lee et al., 2015; Zerbo et al., 2015).

Male sex is a well recognized risk factor for a range of neurodevelopmental disorders including ASDs, early-onset schizophrenia, attention deficit hyperactivity disorder, and learning disabilities (Baron-Cohen et al., 2011; Mandy et al., 2012; Werling and Geschwind, 2013). Despite a preponderance of affected males, females are nevertheless diagnosed with these prevalent disorders in large numbers. Males are at also at elevated risk for generalized complications of pregnancy, showing higher vulnerability to placental inflammation, fetal hypoxia (Eriksson et al., 2010; Sandman et al., 2013), placental abruption, term preeclampsia, eclampsia (Broere-Brown, 2017), and early preterm birth (Challis et al., 2013). Many of these conditions are themselves associated with increased risk of poor neurodevelopmental outcome (Froehlich-Santino et al., 2014; Walker et al., 2015; Ornoy et al., 2016; Straughen et al., 2017). Female fetuses are presumably exposed to the same autosomal genetic and environmental challenges yet generally have better outcomes, with the exception of a higher incidence of fetal growth restriction across populations (Radulescu et al., 2013; Teixeira et al., 2016), suggesting that fetal sex modifies susceptibility to the spectrum of pregnancy complications.

A “female protective effect” has been proposed based on findings that females who are diagnosed with ASD carry a higher burden of inherited genetic risk factors and are more likely to have severely affected siblings (Robinson et al., 2013; Jacquemont et al., 2014). Although this sex bias is well recognized, the causal biology producing sex-dependent vulnerability remains elusive (Mitra et al., 2016). Animal studies have shown that activation of the maternal innate immune system during pregnancy is sufficient to produce neuroanatomical changes with accompanying behavioral alterations in male offspring (Shi et al., 2003; Golan et al., 2005; Meyer et al., 2006; Carpentier et al., 2013; Schaafsma et al., 2017; Custódio et al., 2018; Haida et al., 2019), with females either excluded from these studies or, if included, evaluated solely along parameters originally established in males. (Beery and Zucker 2011; Shansky and Woolley 2016; Coiro and Pollak 2019). An argument is made that males are more likely to be diagnosed with neurodevelopmental disorders, the implication being that an understanding of disrupted development in males is sufficient to elucidate causal mechanisms in a cost-effective manner. Given this practice, it appears to be generally assumed that disease mechanisms and outcomes will be categorically similar with the only distinction being the severity of the effect.

Here, we outline distinct sex-dependent outcomes in mice exposed to a mild maternal immune activation (MIA) elicited by bacterial lipopolysaccharide (LPS) during mid-pregnancy. We and others (Gendron et al., 1990; Fatemi et al., 2012; Eloundou et al., 2019; Mueller et al., 2019) have shown that LPS-induced MIA causes acute vascular damage in the placenta, along with transient hypoxia and reduced neural progenitor cell proliferation in the developing fetal brain (Carpentier et al., 2013). In adulthood, the prenatal insult sharply and specifically reduces the density of Satb2^+^ cells, a population of long-range callosal and commissural projection neurons that begins to appear in cortical plate at mid-gestation (Greig et al., 2013), as well as parvalbumin^+^ (PV) cells, a population of inhibitory interneurons thought to be responsible for setting excitatory/inhibitory balance in forebrain to regulate behavioral responses (Ferguson and Gao, 2018). These changes in cytoarchitecture are accompanied by altered social behavior, repetitive behaviors, and learning deficiencies. We find that while many of these effects are absent or attenuated in females, females show elevation in fetal brain inflammation, altered juvenile social behavior, and an extended delay in adult body growth. Adult females also show a unique MIA-induced increase in PV^+^ neuron density in posterior neocortex and exhibit anxiety-associated behavior not observed in males. Together, these data demonstrate that mild illness during pregnancy is sufficient to cause long-lasting, categorically distinct neuroanatomical and behavioral changes in both male and female mice, illustrating that vulnerabilities underlying the MIA-induced functional alterations can be mutually exclusive between the sexes.

## Materials and Methods

### Experimental design

Timed pregnant C57BL/6J mice were treated with 30 or 60 μg/kg LPS or an equivalent volume of saline at embryonic day 12.5 (E12.5). Outcomes in placenta and fetal brain were measured at 2 and/or 24 h post-treatment, depending on the experiment. Adult outcome measures were determined in animals that were cross-fostered at birth.

#### Animals

All animal studies were performed in accordance with National Institutes of Health (NIH) guidelines for the humane use of animals, and all procedures were reviewed and approved by the Stanford Institutional Animal Care and Use Committee. C57BL/6J mice purchased from The Jackson Laboratory were used for all studies.

#### Timed pregnancies and treatments

To generate timed pregnancies, pairs of females were housed with a single male overnight. Animals were separated the next day, and noon on that day is termed E0.5 for these studies. Pregnancy was confirmed by monitoring postmating weight gain. At E12.5, pregnant mice were injected intraperitoneally with saline or LPS from *Escherichia coli* (Sigma-Aldrich) at 30 or 60 μg/kg body weight prepared in saline. All chemicals were purchased from Sigma-Aldrich except where otherwise noted. For all animals tested postnatally, pups were fostered at birth to wild-type naive mothers with litters between 1 and 5 d old (which were removed). Pups were weaned at 3 weeks of age and group housed at two to five animals per cage in standard microisolator cages with a 12 h light/dark cycle. Animals being analyzed for hypoxia received an intraperitoneal injection of pimonidazole (100 mg/kg; Hypoxyprobe-1) 15 min before saline or LPS.

#### Placental histology

Placentas were dissected and fixed overnight in 10% neutral buffered formalin, followed by dehydration into ethanol, paraffin embedding, and sectioning at 6 μm. H&E staining was performed according to standard protocols (Carpentier et al., 2011). For quantification, images of an entire cross-section were taken at 10× magnification. The spongiotrophoblast layer and the area within the layer characterized by abnormal pink eosinophilic staining were outlined, and pixel areas were determined using ImageJ software version 1.42 (NIH). Data are expressed as the percentage of the spongiotrophoblast layer showing this characteristic staining.

#### Luminex bead array and ELISA

Maternal serum and placentas were harvested 2 h after treatment with saline or LPS (60 μg/kg) at E12.5. Placentas were homogenized in NP-40 lysis buffer with protease inhibitors using a mechanical homogenizer. Total protein was assessed using a protein reagent from Bio-Rad. Cytokine/chemokine levels in the placenta were assessed using a Luminex bead array from Affymetrix according to the manufacturer instructions, with the following incubation times: antibody beads, 2 h at room temperature (RT) followed by overnight at 4°C; detection antibody, 2 h at room temperature; and streptavidin-phycoerythrin, 30 min at room temperature. Standard curves and reports were prepared with MiraiBio MasterPlex QT software (Hitachi Solutions America). Cytokines in placental and fetal brain lysates were measured using ELISA DuoSet kits (R&D Systems), following spike/recovery validation. Placentas and fetal brains were homogenized in lysis buffer with protease inhibitors. The 96-well polystyrene plates were coated with capture antibody overnight at RT, incubated with tissue lysate overnight at 4ºC, incubated with biotinylated detection antibody at RT for 2 h, then detected with strepdavidin and color reagents. Colorimetric measurements were made using a microplate reader (Tecan) set to 405 nm absorbance, and normalized to a 7-point standard curve.

#### Fetal sex genotyping

Sex genotyping was performed on DNA extracted from the fetal body via PCR using primers for X/Y paralog Jarid 1c/d (Forward, 5-CTGAAGCTTTTGGCTTTGAG-3; Reverse, 5-CCGCTGCCAAATTCTTTGC-3), with a strong single 331 bp band connoting XX, and a pair of bands at 331 and 305 bp connoting XY. In examining placental necrosis, DNA was extracted from formalin-fixed and paraffin-embedded placentas using the QIAGEN FFPE DNA Isolation Kit and associated protocol (catalog #56404, QIAGEN).

#### Immunohistochemical analysis in fetal brain

Pimonidazole (Hypoxyprobe-1; HPI) was injected intraperitoneally into pregnant mice at 100 mg/kg body weight 15 min before saline or LPS injection. Two hours later, fetuses were harvested and decapitated. Heads were fixed overnight in 4% paraformaldehyde, equilibrated in 30% sucrose, and rapidly frozen in Fisher Scientific HistoPrep (Thermo Fisher Scientific) in 25.20.5 mm Tissue Tek cryomolds (VWR). Whole heads were sectioned on a Fisher Scientific Microm HM505E Cryostat (Thermo Fisher Scientific) to 16 μm and then were mounted on glass slides. Slides were first rinsed twice with Tris-buffered saline (TBS) and then were blocked in TBS plus 0.3% Triton X-100 plus 10% normal donkey serum (NDS; Jackson ImmunoResearch) for 2 h at room temperature. Tissues were then incubated with primary antibody to pimonidazole (HPI clone 4.3.11.3; 1:50) and phosphohistone H3 (pHH3; 1:400; Cell Signaling Technology) in staining buffer (TBS plus 0.3% Triton X-100 plus 1% NDS) overnight at 4°C. After three washes with TBS, slides were incubated with secondary donkey anti-mouse and donkey anti-rabbit conjugated to Cy3 or fluorescein isothiocyanate (FITC; Jackson ImmunoResearch) diluted 1:500 in staining buffer for 4 h at room temperature. Slides were washed twice in TBS and then incubated with 4′,6′-diamidino-2-phenylindole dihydrochloride (DAPI) at 0.5 μg/ml for 10 min at room temperature. Slides were then washed twice with TBS, fixed with 4% paraformaldehyde for 10 min at room temperature, and washed three more times in TBS. Coverslips were mounted using polyvinyl alcohol and 1,4 diazabicyclo[2.2.2]octane in glycerin.

#### Immunofluorescent staining and antibodies

Adult mice were perfused and fixed with 4% paraformaldehyde. Brains were extracted and equilibrated in 30% sucrose. Adult brains were harvested and sectioned on a freezing sledge microtome. Free-floating brain tissue sections were rinsed twice with TBS and blocked at room temperature in TBS plus 0.3% Triton X-100 and 3–10% normal donkey serum (Jackson ImmunoResearch) for 2 h at room temperature. Tissues were then incubated with primary antibody in staining buffer (TBS plus 0.3% Triton X-100 and 1% NDS; tissue sections) overnight at 4°C. Antibodies included rabbit anti-Satb2 (1:250; Abcam), sheep anti-parvalbumin (1:5000; R&D Systems). After washing 3× with TBS, tissue was incubated with donkey secondary antibodies conjugated to FITC, Cy3, or Cy5 (1:500; Jackson ImmunoResearch) for 4 h at room temperature or overnight at 4°C. Tissue was washed twice in TBS and incubated with DAPI at 0.1 μg/ml for 10 min at room temperature. Tissue was then refixed with 4% paraformaldehyde for 10 min at room temperature and washed, and coverslips were mounted using PVA-DABCO (polyvinyl alcohol and 1,4 diazabicyclo[2.2.2]octane in glycerin).

#### Confocal microscopy, image analysis, and cell counts

For fetal cortex, 20× images were collected on a Zeiss 700 Confocal Microscope with gain and offset adjusted to eliminate undersaturated and oversaturated pixels. Settings were held constant and images were collected from control and experimental tissues of both sexes that had been stained in parallel. For pimonidazole, the area of positive pixels for pimonidazole and DAPI were quantified for each image using identical thresholding criteria. Data are expressed as a ratio of pimonidazole-positive pixels to DAPI^+^ pixels, to normalize for total tissue area within each image frame. The number of pHH3^+^ cells was counted manually and normalized to the brain tissue area using DAPI.

For adult tissue scoring, 40 μm coronal sections were serially collected throughout the entire anterior–posterior axis of the brain. Every sixth section was stained for each marker (96–0 μm sampling interval), and experimental and control tissue from both sexes were stained in parallel in one batch to minimize technical variation in staining. To determine adult cortical projection neuron and interneuron density, cells were scored in six coronal sections, four sections from anterior cortex overlying the lateral ventricles and the genu of the corpus callosum (see Fig. 6*A*, regions I and II) and two sections from posterior cortex overlying the hippocampus (see Fig. 6*A*, region III). The first section in the series was located at the head of the dorsal lateral ventricles, the second section was at the genu of the corpus callosum, and the third section was located at the rostral tip of the hippocampal formation (see Fig. 8*A*). Four-color confocal microscopy was performed with gain and offset adjusted on the brightest and dimmest sections of an entire staining set to prevent undersaturated and oversaturated pixels, ensuring that images could be collected from all animals in a given experiment without changing settings. Images were collected using the 40× oil objective with automated tiling to generate a high-resolution image montage that spanned the entire thickness of the cortex. Slides were blinded to the experimenter, and confocal images were then scored in ImageJ by applying the color threshold tool and “analyze particles” tool. Object area and circularity thresholds for each stain or object type were empirically determined and the accuracy of the automated counts verified by manually scoring a subset of images. Iterative adjustments to image processing, color threshold, and analyze particles variables were made until automated counts were within 3% of manually scored samples. Object area, average pixel intensity, and centroid X and Y coordinates were output to Excel. X and Y coordinates defining the lines of the pial surface and dorsal surface of the corpus callosum were also recorded for each image. The laminar position of a given object was then calculated as a fraction of the distance between pia and corpus callosum.

#### Doppler ultrasound

Umbilical artery blood velocity and fetal heart rate were determined using a Vevo 770 High-Resolution *In Vivo* Micro-Imaging System (VisualSonics) fitted with a 40 MHz transducer. Mice were anesthetized with 3% isoflurane at 2 L O_2_/min for the duration of the scan. Mice were kept warm on a heated platform (37°C) that was connected to a temperature/physiology monitor (Indus Instruments). Abdominal fur was removed using over-the-counter hair remover (Nair, Church & Dwight Co.). Ultrasound Transmission Gel (Parker Laboratories) was applied to the abdominal area. B-mode ultrasound was used to structurally identify placentas, fetuses, and umbilical arteries. The power-Doppler mode was used to monitor blood flow velocities and fetal heart rates. Images were saved as static images or cine loops and quantified using the vendor-provided software.

#### Juvenile play

Dyadic social interactions were measured by using the Juvenile Play paradigm (Silverman et al., 2013). Postnatal day 21 (P21) mice were placed along with a sex- and age-matched stimulus mouse of a different strain (FVB/NJ) into a new cage with clean bedding. Dyadic interactions were recorded for 6 min using an overhead camera and scored manually by blinded observers.

#### Open Field exploration

Open Field is a paradigm that assesses activity, habituation, and anxiety. Mice were habituated to the room for half an hour before testing. A mouse was placed in the center of a 70 × 70 × 50 cm Plexiglas box. The mouse was allowed to explore the box for 20 min. Behavior was recorded using a video camera mounted to the ceiling. The box was cleaned with ethanol after each use. Video files were collected using closed circuit television cameras mounted above the apparatus. The videos were analyzed using MATLAB r2013 with the source code Autotyping 15.03 developed by Tapan Patel at the University of Pennsylvania (Philadelphia, PA; Patel et al., 2014). The Open Field arena surface was determined by manually delineating the boarder of the arena, with software partitions from the arena into inner and outer regions. The outer region is a 2-inch-thick perimeter around the arena floor and the inner region is the area within that perimeter. The software tracked the center of the mouse and computed the amount of time spent in the center region as well as the total distance traveled.

#### Social interaction

Sociability and social memory were tested in an adaptation of the three-chamber test (Crawley, 2007). Briefly, the three-chamber apparatus contains the following three equal-sized chambers: an empty middle chamber and chambers on either end that contain an inverted wire pencil cup, which is used to hold a stimulus mouse. The test has three phases. First, mice were habituated in their home cage to the experimental room for 1 h. Mice were then placed in the testing apparatus and allowed to explore the chambers, with the inverted but empty pencil cups, for 10 min. The subject was then removed, and a novel mouse was placed under one of the pencil cups. The location of the novel mouse was randomized to left and right chambers. The test subject was then placed in the middle chamber, and its activity is recorded for 10 min. For the social memory test, the previously used stimulus mouse (familiar mouse) was placed in the opposite chamber and a new mouse (novel mouse) was placed in the other chamber. The amount of time spent in each chamber and the number of chamber entries were automatically recorded. An experimenter blind to mouse treatment scored the videos to determine the amount of time to initiate contact with the stimulus mouse and the total amount of time spent sniffing empty pencil cups or the stimulus mice using JWatcher software (Bozdagi et al., 2010; Silverman et al., 2013).

#### Marble burying

Marble burying assesses repetitive and stereotyped behavior. We adapted the previously described procedure in the study by Thomas et al. (2009). Mice were habituated to the room for 30 min before testing. Fifteen black glass marbles were place in 5-cm-deep clean bedding in a 3 × 5 arrangement. The mouse was then placed in a test cage for 10 min. Behavior was recorded by a ceiling-mounted camera. After 10 min, the mouse was removed, and the marbles were cleaned with 70% ethanol and dried. Mouse behavior was scored by a blinded experimenter for the treatment by watching the end of the video and counting the number of buried marbles. The criterion for defining a buried marble was over 50% of the marble covered by bedding.

#### Olfactory function

After testing in the social interaction test, mice were tested for olfactory function using a buried food test (Yang and Crawley, 2009). For 2 d preceding the test, mice were given food treats (Froot Loops) in their cages to habituate them to this food. The evening before the test, all food was removed from the cages. The next morning, mice were allowed to habituate to the testing room for 1 h in their home cage. They were then habituated to a new testing cage with 3 cm of bedding for 5 min. Mice were moved to a holding cage while the food was buried under the bedding in the testing cage. Mice were returned to the testing cage, and the amount of time required to find the buried food was recorded by an experimenter blind to treatment group.

#### Delayed match-to-place task

The water maze consisted of a circular black tank (170 cm in diameter, 43 cm deep) filled with water (23–25°C) containing nontoxic tempera paint (Rich Art Color Co.) to obscure the submerged platform (13 cm in diameter, 29 cm in height). Training in the delayed matching-to-place (DMP) task was performed as described previously (Haditsch et al., 2009). The platform was moved to a new location each day, but, within each day, the platform location remained constant. There were 13 possible platform locations, and each was used only once. At the start of each trial, the mouse was gently placed into the water with its head facing the wall of the pool. The start location varied semirandomly between trials (three different starting locations spaced evenly around the pool). The DMP training consisted of six training trials per day with 10–15 min intertrial intervals (ITIs) on days 1–12 and a 1 h ITI on day 13. If a mouse did not find the platform within a 60 s trial, it was placed onto the platform by the experimenter, stayed there for 15 s and then was removed to a warmed home cage. Swim speed, path length, and location data were collected using a video-tracking system (Videotrack Automated Behavioral Analysis System, Viewpoint Life Sciences). Visible platform testing occurred after the DMP task was complete. During the visible platform training, the submerged platform location was indicated by a flag rising above the water line and was visible from all areas within the pool. The experimenter was blind to treatment for all behavioral tests.

### Statistics

Statistical analysis was performed using GraphPad Prism (GraphPad Software). For comparisons between two means, the two-tailed Student’s *t* test was used. For comparisons in assays that varied by two variables, two-way ANOVA was performed with Tukey’s post-tests. For the delayed DMP and open field, repeated-measures ANOVA was performed to compare groups across sequential trials, time points, or days. The mean ± SEM values are presented in all figures (Table 2, summary of statistics).

## Results

### LPS-induced maternal cytokines evoke more focal hemorrhage and greater impairments in placental growth in males

**Figure 1.**
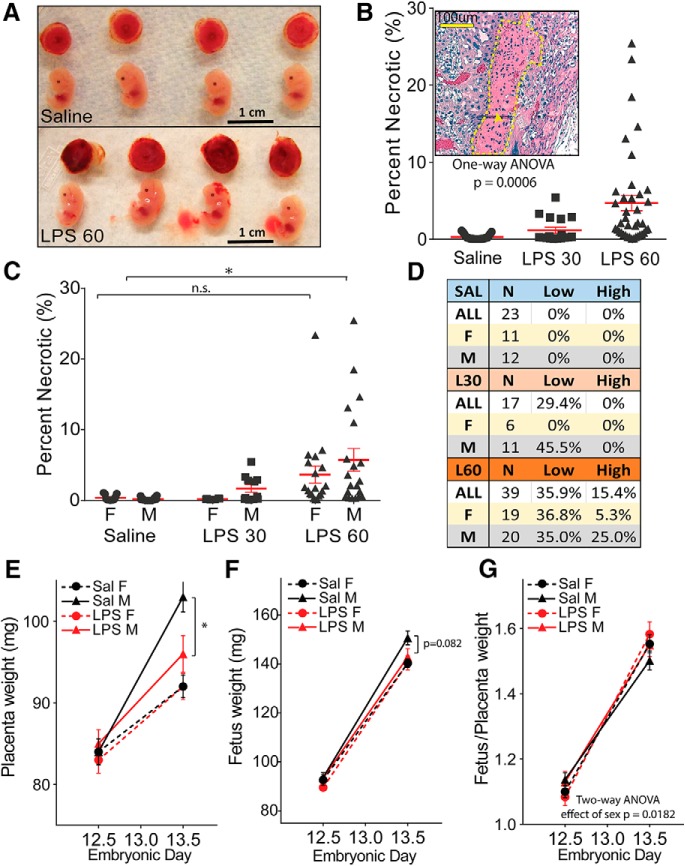
Male-selective placental necrosis and loss of growth potential. ***A***, Representative fetus and placenta 2 h following saline or LPS (60 μg/kg) treatment. ***B***, Placentas were collected from surviving fetuses 24 h after E12.5 saline or LPS treatment (Extended Data Fig. 1-1), formalin-fixed, and H&E stained to identify necrosis (inset). Tracts of eosinophilic necrotic tissue in the spongiotrophoblast layer are outlined by the yellow dotted line. Scale bar, 100 μm. Areas of necrotic tissue were measured as a percentage of total spongiotrophoblast layer area in pregnancies treated with saline, LPS 30 μg/kg, and LPS60 μg/kg. ***C***, The same percentage of necrosis data plotted by fetal sex. F, Female; M, male. ***D***, Necrosis scores binned into low-damage (2–8%) and high-damage (>8%) categories. ***E***, ***F***, Placentas (***E***) and fetuses (***F***) were weighed immediately after dissection at 2 and 24 h post-treatment with saline or LPS (60 μg/kg). ***G***, The ratio of fetal/placental weight was compared. (mean ± SEM). Grouped necrosis scores compared by one-way ANOVA, and sex-specific necrosis compared using a two-way ANOVA with appropriate *post hoc* tests. Weights and ratios compared by two-way ANOVA. *p < 0.05, n.s. = not significant.

To investigate the effect of maternal inflammation on the placenta, a maternal innate immune response was elicited by intraperitoneal injection of 60 µg/kg LPS at E12.5. The maternal cytokines elicited by this dose were sufficient to cause small focal hemorrhage and tracts of tissue necrosis within the placenta but sufficiently mild to allow all pregnancies to progress to term (Carpentier et al., 2011). At 2 h after LPS injection, regional hemorrhage was observed at the perimeter of the placenta (Fig. 1*A*). By 24 h, the vascular congestion had diminished, and the placentas showed prominent tracts of tissue necrosis in the spongiotrophoblast layer, highlighted by eosin staining and pyknotic nuclei (Fig. 1*B*). Female placentas were significantly more resilient. Significant tissue necrosis was observed in a subset of males at the lowest dose tested (30 μg/kg; Fig. 1*C*,*D*), while placentas of female littermates were unaffected. At a dose of 60 μg/kg, male placentas incurred significantly more damage than those in controls (two-way ANOVA with Tukey’s *post hoc* test, *p* = 0.0161), while the level of necrotic damage in female placentas resembled those in males from the lower dose, suggesting that male placentas have an accentuated vulnerability to cytokine-evoked bleeding and tissue necrosis.

10.1523/ENEURO.0358-19.2019.f1-1 Figure 1-1 Fetal loss and sex ratios 24 h after LPS. ***A***, Proportion of live and dead fetuses 24 h after saline or LPS 60 μg/kg treatment were determined by gross examination. Blanched and resorbing fetuses were counted as dead, while pink and intact fetuses were considered alive at the time of dissection. ***B***, Sex ratio of surviving fetuses was determined by PCR genotyping of Jarid1c/d in fetal tissue (*p* values from Fisher’s exact test). Download Figure 1-1, TIF file.

Placental and fetal growth were also examined. Under control conditions, female placentas gain significantly less weight between E12.5 and E13.5 than male placentas (*p* < 0.0001, two-way ANOVA, effect of sex). At 24 h after LPS treatment, female placental growth was unaffected while male placentas showed a significant reduction in growth compared with control males (two-way ANOVA, effect of treatment in males, *p* = 0.0182; Fig. 1*E*). Female fetal growth was also entirely unaffected by LPS treatment (two-tailed *t* test, *p* = 0.9662), while growth of the male fetus showed a trend toward decreased fetal body weight after LPS treatment (Fig. 1*F*; two-tailed *t* test, *p* = 0.0818). The ratio of fetal/placental weight was higher in females at E13.5, regardless of treatment (Fig. 1*G*).

### Fetal loss is not affected by sex

The placental injury was also accompanied by a dose-dependent loss of fetuses in each pregnancy (Carpentier et al., 2011). Treatment with 60 μg/kg LPS at E12.5 caused an average of 21% of fetuses to die and begin to be resorbed (Extended Data Fig. 1-1*A*). In contrast with the elevated placental injury in males, the decrease in male survival did not reach significance (Extended Data Fig. 1-1*B*).

### Proinflammatory cytokines are more highly elevated in male placenta

**Figure 2.**
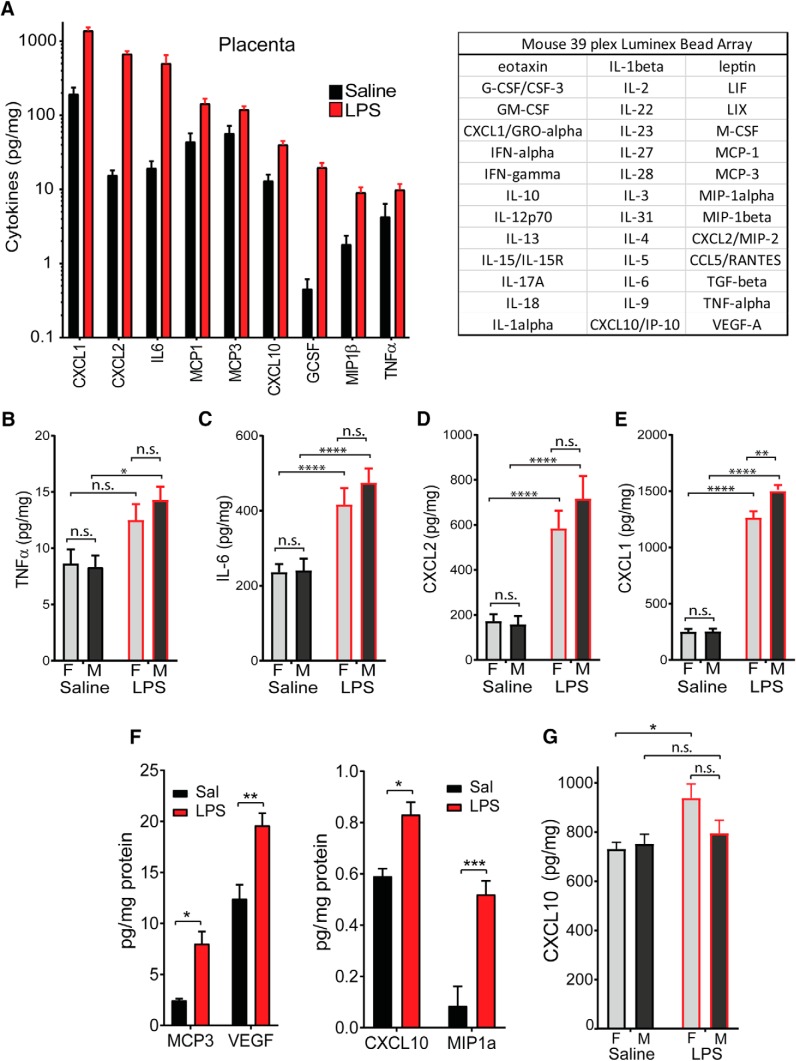
Cytokines spikes in placenta and fetal brain at 2 h. ***A***, Placentas were collected at 2 h after treatment and were analyzed for cytokine abundance using a 39-plex mouse Luminex bead array (table). The specific cytokines depicted were determined to be significantly elevated by two-tailed *t* test (*p* < 0.05). Data are plotted as picograms cytokine/milligram of total protein. ***B–E***, An expanded set of placenta lysates was assayed for cytokine and chemokine abundance by ELISA TNFα (***B***), IL-6 (***C***), CXCL2 (***D***), and CXCL1 (***E***). ***F***, ***G***, Fetal brains of unknown sex were collected at 2 h after treatment and were analyzed for cytokine abundance using the same 39-plex mouse Luminex panel. The cytokines depicted were determined to be significantly elevated by two-way ANOVA (*p* < 0.05), with a higher abundance (left) and a lower abundance (right) of cytokines depicted. ***G***, Abundance of CXCL10 was measured by ELISA in a separate group of fetal brain lysates and compared by sex (data are plotted as picograms cytokine/milligram of total protein; mean ± SEM. ELISA groups compared by two-way ANOVA with appropriate *post hoc* tests: *p < 0.05, **p < 0.01, ***p < 0.001, ****p < 0.0001, n.s. = not significant.

To examine the relationship between placental damage and inflammatory signaling, we evaluated cytokine abundance in the placenta after LPS injection (60 μg/kg) using Luminex multiplex assays. Levels of CXC ligand (CXCL) 1, CXCL2, IL-6, monocyte chemoattractant protein (MCP)-1, MCP-3, CXCL10, GCSF (granulocyte-colony-stimulating factor), MIP-1β (macrophage inflammatory protein 1β), and TNFα were identified as being significantly elevated in placental lysates assayed blind to sex (*p* < 0.05, two-tailed *t* test; Fig. 2*A*). To more precisely examine sex differences in TNFα and IL-6, cytokines that are known to produce pathology in MIA models (Carpentier et al., 2011), as well as CXCL1 and CXCL2, chemokines that are by far the most abundant in the inflamed placenta at 2 h, an expanded set of placental lysates was examined using ELISA. TNFα level was significantly elevated in the male placenta 2 h after LPS (Fig. 2*B*; two-way ANOVA with Tukey’s *post hoc* test), but not in the female placenta. Overall, cytokine levels trended toward being more elevated in male placenta than in female placenta, especially for abundant cytokines, such as IL-6 and CXCL2 (Fig. 2*C–E*). This elevation reaches significance for CXCL1, the chemokine that shows the highest abundance in the placenta at 2 h after LPS injection, with levels in males reaching a higher spike than females (Fig. 2*E*; two-way ANOVA: *p* = 0.0106 for effect of sex; *p* < 0.0001 for treatment; and *p* = 0.0122 for interaction), suggesting a general trend toward heightened acute inflammation in the male placenta.

### Chemotactic cytokine CXCL10 is elevated in female brain at 2 h following LPS injection

The placenta is the barrier that protects the developing fetus from the maternal immune system. When the placenta itself is inflamed, it is possible that this protection breaks down and tissues like the developing fetal brain could be aberrantly exposed to inflammatory mediators, leading to alterations in development and behavior. To determine whether inflammatory signaling is acutely elevated in fetal brain following maternal LPS exposure, a Luminex assay of fetal brain lysate was performed to identify cytokines having elevated levels in fetal brain 2 h after LPS injection (Fig. 2*F*). MCP-3, MIP-1α, VEGF, and CXCL10 were identified as being elevated (two-way ANOVA, Tukey’s *post hoc* test, *p* < 0.05). When more closely analyzed by fetal sex using ELISA, CXCL10 level was found to be elevated exclusively in the female brain (Fig. 2*G*; two-way ANOVA for significant effect of LPS, saline female vs LPS female, *p* = 0.0207 by Tukey’s *post hoc* test).

### Hypoxia in the fetal cortex is more severe in males

**Figure 3.**
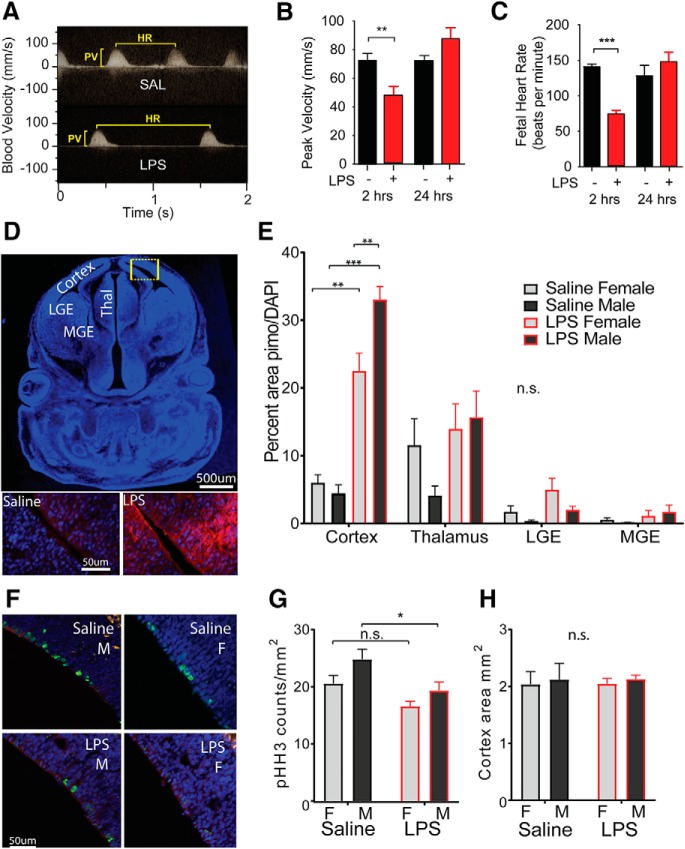
Hypoxia and reduction in progenitor proliferation are more pronounced in male fetuses. ***A***, Doppler ultrasound was used to measure umbilical artery blood flow 2 or 24 h after saline or LPS treatment on E12.5. A peak of the Doppler velocity plot represents a fetal heart beat, and the height of the peak represents the peak velocity of the blood flow. ***B***, Quantification of peak velocity in the umbilical artery in saline- and LPS-treated mothers 2 or 24 h post-treatment. ***C***, Quantification of fetal heart rates in beats per minute for saline- and LPS-treated pregnancies 2 or 24 h after treatment. ***D***, The proportion of hypoxic fetal brain tissue was estimated by treating dams with pimonidazole 15 min before treatment with LPS (60 μg/kg) or saline at E12.5. Two hours after treatment, fetuses were harvested and coronal brain sections were stained for pimonidazole adducts, which indicate previous exposure to hypoxic conditions (<1.5% oxygen). Scale bar, 50 μm. ***E***, Hypoxia was quantified in fetal cortex, thalamus, LGE, and MGE as the proportion of DAPI^+^ tissue (blue) positive for pimonidazole (red) in uniformly thresholded images. ***F***, The same fetal brain sections were also stained for M-phase marker pHH3 (green). ***G***, Quantification of pHH3^+^ nuclei per square millimeter of cortical tissue. ***H***, Average area of fetal cortex measured per condition (in square millimeters). Mean ± SEM; ultrasound groups were compared by two-tailed *t* tests. All other groups compared by two-way ANOVA with appropriate *post hoc* tests: *p < 0.05, **p < 0.01, ***p < 0.001, n.s. = not significant.

We have previously shown that maternal LPS treatment transiently diminishes the oxygen supply to the developing fetal brain (Carpentier et al., 2011, 2013). To determine whether placental transport and fetal perfusion are impaired in a manner that would affect oxygen supply to the fetal brain, we evaluated pregnant mice for changes in fetal heart rate and umbilical blood flow using Doppler microultrasound (Fig. 3*A*). We found that peak blood velocity and fetal heart rate were significantly reduced within 2 h of LPS administration (Student’s two-tailed *t* test: blood velocity, *p* = 0.01; heart rate, *p* < 0.0001). However, the effects were transient, with both velocity and heart rate restored by 24 h after LPS challenge in surviving pups (Fig. 3*B*,*C*). Although it was not possible to determine the sex of the fetus by ultrasound, we were able to inject pregnant animals with pimonidazole, a compound that can cross the placenta and form adducts with proteins in hypoxic tissues (Carpentier et al., 2011) and then collect and evaluate brains from male and female fetuses. Pimonidazole (100 mg/kg, i.p.) was injected 15 min before injection of saline or LPS (60 μg/kg) to equilibrate within maternal and fetal tissues, and fetal brain was then collected 2 h after LPS injection. Coronal sections were evaluated with immunofluorescent staining to detect pimonidazole in the cortex, thalamus, median ganglionic eminence (MGE) and lateral ganglionic eminence (LGE; Fig. 3*D*). LPS challenge in the mother resulted in a significant increase in the fraction of pimonidazole-labeled hypoxic tissue in cortex, thalamus, and LGE (Fig. 3*E*). Of these regions, the cortex displayed the largest increase in pimonidazole staining, and this increase was significantly more extensive in male cortex than in female cortex (two-way ANOVA: *p* = 0.0491 for effect of sex; *p* < 0.0001 for treatment; and *p* = 0.0104 for interaction; LPS male vs LPS female, *p* = 0.0081 by Tukey’s *post hoc* test).

### Radial glia proliferation is not significantly affected in females but undergoes an acute depression in male fetal cortex

To determine whether the hypoxic challenge produced by MIA impacts the process of corticogenesis, fetal brains were collected at 2 h post-treatment and frozen sections were stained for pHH3^+^, a marker for cells in metaphase (Fig. 3*F*). We have previously reported that the number of pHH3^+^ radial glia located at the ventricular surface are reduced in the fetal brain during the hypoxic challenge (Carpentier et al., 2011, 2013). When animals were reanalyzed by sex, males showed slightly higher baseline proliferation and a more profound reduction in metaphase cells (Fig. 3*G*; two-way ANOVA: *p* = 0.0188 for effect of sex; *p* = 0.0021 for effect of treatment) with LPS producing a significant decrease (*p* = 0.0414 by Tukey’s *post hoc* test). Females showed a slightly lower baseline of proliferation and a nonsignificant trend toward reduction with LPS. There was no between-group difference in fetal cortex area by two-way ANOVA, suggesting that the developmental stages of all groups were roughly matched (Fig. 3*H*).

### Maternal immune activation delays postnatal body growth in female offspring

**Figure 4.**
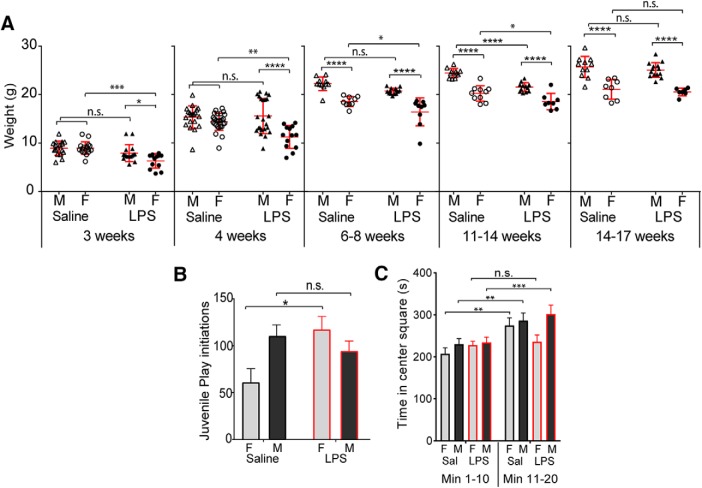
Female-selective postnatal growth delay and behavioral outcomes. Pregnant mice were treated with saline or LPS (60 μg/kg) at E12.5. At birth, offspring were fostered to naive mothers. ***A***, Animals were weighed at 3 and 4 postnatal weeks, and again at 6–8, 11–14, and 14–17 postnatal weeks, and weights were compared by two-way ANOVA for each age group. ***B***, At P21, juvenile animals were paired with a stimulus mouse of a different strain, and social interaction initiations were observed for 6 min. ***C***, On an open field task of exploration and anxiety, mice were allowed to explore an open chamber with an unsheltered central area. Time in the inner area was recorded along with total ambulation (Extended Data Fig. 4-1), presented as early (0–10 min) and late (10–20 min) phases across both trials. Weights are depicted as mean ± SD; juvenile play and open field are depicted as the mean ± SEM. Groups compared by two-way ANOVA with appropriate *post hoc* tests for adult weights and juvenile play, repeated-measures ANOVA with *post hoc* test was used for open field. *p < 0.05, **p < 0.01, ***p < 0.001, ****p < 0.0001, n.s. = not significant.

Earlier work shows that MIA offspring are smaller in adulthood than are saline controls (Carpentier et al., 2013). When analyzed by sex, we find that this extended effect is pronounced in females. Body weights begin to diverge between LPS- and saline-exposed female offspring at 3 postnatal weeks, while males remain unaffected (Fig. 4*A*; two-way ANOVAs performed for each time point). This female-selective growth delay remained pronounced at postnatal week 4, persisting through 6–8 postnatal weeks and 11–14 weeks. Males exhibited a transient growth delay between 11 and 14 weeks, and both sexes were normalized in weight by 14–17 postnatal weeks, which is consistent with normal adult sexual dimorphism, without any further impact of MIA.

10.1523/ENEURO.0358-19.2019.f4-1 Figure 4-1 Activity, social novelty, and olfaction. Some behavioral tests revealed subtle or no sex differences. ***A***, On an open field task of exploration and anxiety, mice were allowed to explore an open chamber with an unsheltered central area. Total ambulation time over the course of the trial, presented as early and late 10 min phases. ***B***, Subsequent to the three-chamber social interaction test, mice were exposed to a novel mouse under the previously empty cup, and preference is reported as the percentage of time spent interacting with the novel mouse over the familiar mouse, an indicator of social memory. ***C***, The open field cohort underwent a buried food test, and the time to find cereal buried in cage bedding was recorded. Comparisons were made by two-way ANOVA for social memory and buried food, and repeated-measures ANOVA with *post hoc* for ambulation: **p < 0.01, ***p < 0.001, n.s. = not significant. Download Figure 4-1, TIF file.

### Females exhibit altered juvenile interaction and thigmotaxis during exploration

At P21, dyadic social interactions were measured using the juvenile play paradigm (Silverman et al., 2013). Pups were placed with a sex- and age-matched stimulus pup of a different coat color to facilitate scoring of experimental and stimulus animals (FVB/NJ). Social interaction initiations were observed for a period of 6 min. There was no overall effect of MIA on social behavior independent of sex (Fig. 4*B*) and no effect of sex independent of treatment. However, there was a significant interaction between treatment and sex, with MIA females initiating significantly more interactions than saline control females. MIA males showed a slight but nonsignificant opposite effect with a decrease in social initiations compared with saline control males.

Adult animals were assayed for exploratory behavior using an open field task (Patel et al., 2014) in which mice are allowed to explore a walled open chamber. Mice instinctively avoid the exposed center region but gradually habituate and begin to explore more thoroughly. MIA females did not show the expected habituation to the open field chamber and failed to increase exploration of the center field, while all other groups became more exploratory in the center of the open field (repeated-measures ANOVA with *post hoc* test, effect of trial phase, *p* < 0.0001; Fig. 4*C*). Saline-exposed control animals of both sexes showed the expected decrease in ambulation in the latter phase of the trial, while MIA animals of both sexes did not (Extended Data Fig. 4-1*A*).

### Alterations in adult social behavior are observed only in males

We have previously demonstrated that prenatal MIA produces impairments in social behavior, learning, and memory that are significant in males but not in females (Carpentier et al., 2013). Here, we performed a more comprehensive assessment of sex differences in behavior to determine the extent of male-selective behavioral abnormalities.

**Figure 5.**
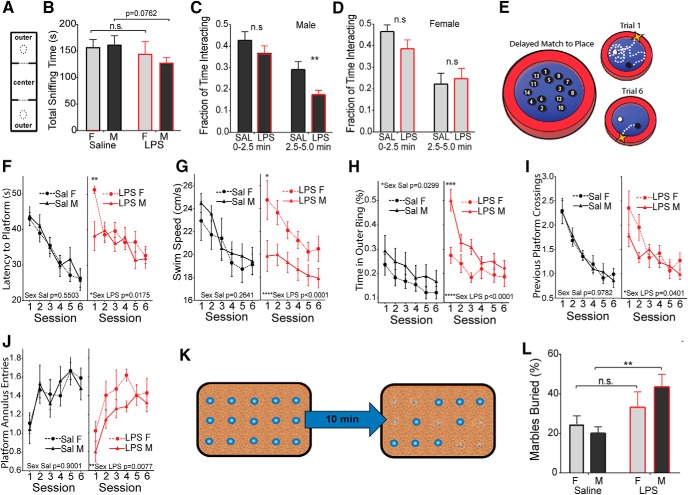
Male-selective decrease in socialization and working memory, and an increase in stereotypies. Pups from E12.5 challenged pregnancies (LPS vs saline) were fostered and aged to 4 weeks. ***A***, ***B***, Animals were placed in a three-chamber compartment with an empty cup, a central chamber, and a cup containing a stimulus mouse of matched sex (***A***), and total interaction time (sniffing) was recorded for 10 min (***B***). ***C***, ***D***, Time interacting with the stimulus mouse was recorded as a fraction of total time at the early and middle phases of the trial for males (***C***) and females (***D***). ***E***, Adult animals (6–8 weeks) were tested for impairments in working memory using the DMP water maze task. ***F–J***, Latency to find the platform was recorded over six sessions of two trials per session (***F***), along with swim velocity in centimeters per second (***G***), thigmotaxis (perimeter circling; ***H***), crosses into the previous platform area (***I***), and entries into the current platform location (***J***). ***K***, Schematic of the marble-burying test. ***L***, Fraction of buried marbles was recorded after 10 min (mean ± SEM). Comparisons were made by two-tailed *t* test for total socialization (***A***), repeated-measures ANOVA with *post hoc* test for DMP tests (***G–K***), and two-way ANOVA was used for all other measures with appropriate *post hoc* tests. *p < 0.05, **p < 0.01, ***p < 0.001, ****p < 0.0001, n.s. = not significant.

Social interaction and preference for social novelty were tested using an adaptation of the three-chamber social interaction paradigm (Crawley, 2007), as described previously (Carpentier et al., 2013). In the sociability phase of testing, a stimulus mouse was placed under a wire pencil cup in one of the outer chambers, and the test subject was allowed to explore all three chambers for 10 min (Fig. 5*A*). Male mice from MIA pregnancies showed a trend toward spending less total time interacting with the stimulus mouse (Fig. 5*B*). Mice display a rapid habituation to the presence of a novel stimulus mouse that is most pronounced in the first 5 min of the test. Examination of interactions during the 0–2.5 min and 2.5–5 min phases of the trial revealed a significant sex bias, with MIA males losing sex-typical interest in the stimulus mouse more quickly than controls (Fig. 5*C*), while sex-typical female behavior was not significantly affected (Fig. 5*D*). In the preference for the social novelty phase of testing, the original stimulus mouse is placed under the cup in one chamber and a novel mouse is placed under the cup in the opposite chamber. Saline and LPS treatment groups both preferred interacting with the novel mouse, and there was no significant difference between groups (Extended Data Fig. 4-1*B*). Social behavior in mice relies on intact olfaction. A buried food test conducted in the same cohort confirmed that olfactory function was not impaired by MIA (Extended Data Fig. 4-1*C*).

### Males show impairments on delayed match to place task in the Morris water maze

A separate cohort of mice was tested for alterations in associative processing, learning, and memory in the DMP water maze task (Haditsch et al., 2009). In the DMP task, a hidden escape platform is placed in a new location on consecutive training days. Each day, animals must first locate the platform and then remember its location in the subsequent trials (Fig. 5*E*). Both male and female controls and MIA offspring were equally adept at learning the task, but the latency to find the platform in session 1 of each day was significantly larger in MIA females (Fig. 5*F*). Female and male controls show similar swim speeds, while MIA males show a pronounced decrease in swim speed relative to MIA females (Fig. 5*G*). MIA males also show a substantial increase in thigmotaxis (perimeter-circling behavior; Fig. 5*H*). The platform location is moved for the trials of each day, and animals will naturally return to the location of the previous day. MIA females showed an unimpaired memory of the platform location of the previous day, while MIA males made fewer crossings of the previous platform location during the first session of the day, indicating impaired memory of the platform position of the previous day (Fig. 5*I*). Prenatal LPS exposure also produced a sex difference in the entries into an annulus around the current platform location (swimming near the platform). MIA females were unaffected, while MIA males showed a significant decrease in annulus entries, indicating that males are impaired both in remembering the previous platform location and in learning the new location (Fig. 5*J*).

### Males show increased repetitive behaviors

Mice were also observed for signs of repetitive or stereotypic behavior using a marble-burying task (Thomas et al., 2009), in which 15 marbles were placed on top of the cage bedding and the proportion of marbles buried by the mouse was recorded at the end of the 10 min trial period (Fig. 5*K*). Both females and males show an increase in the mean number of marbles buried but the amplitude of the change is only significant in males, suggesting a male-biased increase in behavioral stereotypies (Fig. 5*L*; two-way ANOVA for effect of treatment, saline male vs LPS male, *p* = 0.0028 by *post hoc* test).

### There are sex-dependent changes in Satb2 and PV neuron abundance and laminar positioning in adult cortex

The patterning of neuronal subtypes ventricular zone changes over time to generate distinct cellular populations in each layer (Molyneaux et al., 2007). The number, density, and laminar location of these populations are likely to impact both cell function and overall circuit-level organization. Accordingly, cortical tissues were evaluated in the following two ways: tissues were first scored for cell density in all lamina and then scored for the distribution of cells among 10 equally distributed laminar zones or “bins” (laminar distribution). We have previously reported that while overall brain size is normal in adult animals exposed to MIA, there were several stereotypical alterations in the laminar patterning of the neocortex in these animals (Carpentier et al., 2013). In this previous work, we evaluated numerous neuronal subtype markers and found that many cell types were unaffected by MIA with the exception of a highly significant depletion of the population of Satb2^+^ corticocortical projection neurons throughout the anterior cortex and a parallel depletion of PV-expressing interneurons, a large population of fast-spiking inhibitory neurons operating within local circuits that engage Satb2^+^ projection neurons (Table 1). However, sex was not initially considered as a variable in these experiments.

**Table 1.** Summary of cortical patterning differences after E12.5 LPS

Cell density cells/mm^2^	DAPI						Satb2						Parvalbumin					
	Counts			ANOVA			Counts			ANOVA			Counts			ANOVA		
	Saline	LPS		Treatment	Interaction		Saline	LPS		Treatment	Interaction		Saline	LPS		Treatment	Interaction	
All layers (bins 1–10)	M	9642.32	9123.68	*p*	0.237	0.867	M	2707.93	1537.57	*p*	**<0.001**	0.239	M	1209.02	1041.96	*p*	**0.020**	0.957
SD	2958.59	1846.88	*F*	1.42	0.5	SD	1014.05	632.75	*F*	88.05	1.33	SD	656.23	458.25	*F*	5.65	0.35	
Superficial layers (bins 1–5)	M	9781.21	8888.16	*p*	0.1815	0.9483	M	2487.85	1468.41	*p*	**<0.001**	0.1688	M	1218.28	1082.06	*p*	0.2471	0.8386
SD	3089.57	1678.11	*F*	1.85	0.18	SD	1270.08	792.36	*F*	29.22	1.71	SD	695.57	407.22	*F*	1.38	0.36	
Deep layers (bins 6–10)	M	9503.43	9359.21	*p*	0.802	0.491	M	2928.00	1606.73	*p*	**<0.001**	0.553	M	1199.77	1001.86	*p*	**0.017**	0.820
SD	2878.48	2008.09	*F*	0.06	0.09	SD	629.00	423.83	*F*	65.50	0.77	SD	628.67	509.47	*F*	6.20	0.38	
	Tbr1						Cux1						GABA					
	Counts			ANOVA			Counts			ANOVA			Counts			ANOVA		
	Saline	LPS		Treatment	Interaction		Saline	LPS		Treatment	Interaction		Saline	LPS		Treatment	Interaction	
All layers (bins 1-10)	M	3307.61	2418.87	*p*	**0.003**	0.695	M	447.82	356.42	*p*	**0.034**	0.991	M	425.72	488.07	*p*	0.286	0.924
SD	2784.79	2082.29	*F*	9.43	0.71	SD	312.45	243.69	*F*	4.62	0.22	SD	243.19	343.94	*F*	1.16	0.42	
Superficial layers (bins 1–5)	M	3801.95	3106.26	*p*	0.1613	0.9301	M	621.21	525.03	*P*	0.1817	0.9459	M	431.30	565.78	*p*	0.1396	0.9094
SD	3237.13	2388.12	*F*	2.04	0.21	SD	307.00	235.83	*F*	1.85	0.18	SD	231.67	405.55	*F*	2.27	0.26	
Deep layers (bins 6–10)	M	2813.28	1731.47	*p*	**0.001**	0.106	M	274.43	187.81	*p*	0.074	0.823	M	420.13	410.37	*p*	0.896	0.876
SD	2201.15	1471.40	*F*	12.00	2.05	SD	206.01	80.01	*F*	3.37	0.38	SD	258.85	253.89	*F*	**0.02**	0.30	

M, Mean. Bold type indicates significance. Two-way ANOVA for treatment and interaction effects on the cell densities in laminar bin. Significance was calculated across all bins or calculated separately for the subset of 5 bins located in superficial or deep layers of the cortex subsets of cortical projection neurons. Cux1 serves as a marker for upper layer neurons. GABA serves as a marker for cortical interneurons, and parvalbumin marks a major subset of interneurons.

**Table 2.** Summary of sample sizes, litter numbers, and statistical tests by figure

Figure	Panel (group)	M	F	Litter	Measure	Test	Comparison	df	Statistic	*p* Value
Figure 1	*A–C* (saline)	12	11	9	(A) Placental necrosis	One-way ANOVA	Effect of LPS treatment	2, 76	*F* = 8.15	**0.0006**
	*A–C* (LPS 30)	11	9	5		One-way ANOVA	Saline vs LPS30 (Tukey's *post hoc* test)	76	*q* = 0.8648	0.8143
	*A–C* (LPS 60)	20	19	16		One-way ANOVA	Saline vs LPS60 (Tukey's *post hoc* test)	76	*q* = 5.279	**0.0010**
						One-way ANOVA	LPS30 vs LPS60 (Tukey's *post hoc* test)	76	*q* = 3.823	**0.0228**
					(B, C) Placental necrosis	Two-way ANOVA	Effect of LPS treatment	2, 73	*F* = 8.16	**0.0006**
						Two-way ANOVA	LPS60 vs saline males (Tukey’s *post hoc* test)	73	*q* = 4.722	**0.0161**
						Two-way ANOVA	LPS60 vs saline females (Tukey’s *post hoc* test)	73	*q* = 2.7	0.4051
	*D–F* (Sal 2 h)	65	57	18	(D) Placental weight	Two-way ANOVA	Effect of sex E13.5	1, 230	*F* = 17.65	**<0.0001**
	*D–F* (Sal 24 h)	76	66	22		Two-tailed *t* test	LPS effect in males E13.5	108	*t* = 2.398	**0.0182**
	*D–F* (LPS 2 h)	31	41	10		Two-tailed *t* test	LPS effect in females E13.5	122	*t* = 0	>0.9999
	*D–F* (LPS 24 h)	48	44	18	(E) Fetal weight	Two-way ANOVA	Effect of sex E13.5	1, 286	*F* = 5.343	**0.0215**
						Two-tailed *t* test	LPS effect in males E13.5	134	*t* = 1.753	0.0818
						Two-tailed *t* test	LPS effect in females E13.5	152	*t* = 0.0425	0.9662
					(F) Ratio placenta/fetal weight	Two-way ANOVA	Effect of sex E13.5	1, 230	*F* = 17.65	**0.0182**
Figure 2	*A* (all groups)	6	6	3	(A) Placenta cytokine abundance	Two-way ANOVA	Overall effect of treatment	1, 90	*F* = 124.9	**<0.0001**
						Multiple *t* test	Each elevated cytokine	10	*q* < 0.01	**<0.05**
	*B–E* (all groups)	8	8	4	(B) TNFα abundance	Two-way ANOVA	Effect of LPS treatment	1, 34	*F* = 14.82	**0.0005**
						Two-way ANOVA	Effect of LPS in males (Tukey’s *post hoc* test)	34	*t* = 3.302	**0.0135**
						Two-way ANOVA	Effect of LPS in females (Tukey’s *post hoc* test)	34	*t* = 2.143	0.2143
					(E) CXCL1 abundance	Two-way ANOVA	Effect of LPS treatment	1, 34	*F* = 655.2	**<0.0001**
						Two-way ANOVA	Effect of sex	1, 34	*F* = 7.322	**0.0106**
						Two-way ANOVA	Interaction	1, 34	*F* = 7.013	**0.0122**
	*F, G* (All groups)	3 fetuses		3	(F) Brain Luminex cytokines (High set)	Two-way ANOVA	Overall effect of LPS	1, 8	*F* = 34.31	**0.0004**
		sex unknown				Two-way ANOVA	LPS effect on MCP3 (Tukey's *post hoc* test)	8	*t* = 3.614	**0.0136**
						Two-way ANOVA	LPS effect on VEGF (Tukey's *post hoc* test)	8	*t* = 4.669	**0.0032**
					(G) Brain Luminex cytokines (Low set)	Two-way ANOVA	Overall effect of LPS	1, 8	*F* = 38.65	**<0.0001**
						Two-way ANOVA	LPS effect on MIP1α (Tukey's *post hoc* test)	8	*t* = 3.128	**0.0009**
						Two-way ANOVA	LPS effect on CXCL10 (Tukey's *post hoc* test)	8	*t* = 5.664	**0.0279**
	*H* (saline)	6	6	3	(H) CXCL10 abundance (ELISA)	Two-way ANOVA	Effect of LPS treatment	1, 20	*F* = 7.532	**0.0125**
	*H* (LPS)	6	6	3		Two-way ANOVA	Effect of LPS in females (Tukey's *post hoc* test)	20	*q* = 4.55	**0.0207**
						Two-way ANOVA	Effect of LPS in males (Tukey's *post hoc* test)	20	*q* = 0.9392	0.9093
Figure 3	*B, C* (All groups)	9 fetuses		>3	(B) Umbilical perfusion	Two-tailed *t* test	Saline vs LPS 2 h	35	*t* = 4.096	**0.0100**
		sex unknown				Two-tailed *t* test	Saline vs LPS 24 h	35	*t* = 1.368	0.0900
					(C) Fetal heart rate	Two-tailed *t* test	Saline vs LPS 2 h	47	*t* = 5.970	**<0.0001**
						Two-tailed *t* test	Saline vs LPS 24 h	47	*t* = 0.307	0.3800
	*E* (saline)	6	5	3	(E) Hypoxia (all regions)	Two-way ANOVA	Effect of LPS in males	1, 36	*F* = 90.47	**<0.0001**
	*E* (LPS)	5	7	4		Two-way ANOVA	Effect of LPS in females	1, 34	*F* = 9.128	**0.0048**
						Two-way ANOVA	Effect of LPS in cortex (both sexes)	1, 18	*F* = 113.5	**<0.0001**
						Two-way ANOVA	Effect of sex in cortex	1, 18	*F* = 4.455	**0.0491**
						Two-way ANOVA	Interaction cortex	1, 18	*F* = 8.186	**0.0104**
						Two-way ANOVA	LPS M vs LPS F cortex (Tukey's *post hoc* test)	18	*q* = 5.232	**0.0081**
						Two-way ANOVA	Effect of LPS in thalamus (both sexes)	1, 17	*F* = 3.844	0.0665
						Two-way ANOVA	Effect of sex in thalamus	1, 17	*F* = 0.640	0.4348
						Two-way ANOVA	Interaction thalamus	1, 17	*F* = 1.650	0.2162
						Two-way ANOVA	Effect of LPS in LGE (both sexes)	1, 20	*F* = 3.553	0.0741
						Two-way ANOVA	Effect of sex in LGE	1, 20	*F* = 4.502	**0.0465**
						Two-way ANOVA	Interaction LGE	1, 20	*F* = 0.496	0.4893
						Two-way ANOVA	Effect of LPS in MGE (both sexes)	1, 15	*F* = 2.438	0.1393
						Two-way ANOVA	Effect of sex in MGE	1, 15	*F* = 0.016	0.9011
						Two-way ANOVA	Interaction MGE	1, 15	*F* = 0.545	0.4717

	*G* (saline)	9	9	5	(G) Metaphase cells in VZ	Two-way ANOVA	Effect of LPS treatment	1, 34	*F* = 11.07	**0.0021**
	*G* (LPS)	11	9	6		Two-way ANOVA	Effect of sex	1, 34	*F* = 6.092	**0.0188**
						Two-way ANOVA	Effect of LPS in males (Tukey's *post hoc* test)	34	*q* = 3.935	**0.0414**
					Two-way ANOVA	Effect of LPS in females (Tukey's *post hoc* test)	34	*q* = 2.749	0.2295	
					(H) Total cortex area	Two-way ANOVA	Effect of sex on cortex area	1,20	*F* = 0.2975	0.5915
						Two-way ANOVA	Effect of LPS on cortex area	1,20	*F* = 0.0043	0.9486
Figure 4	*A* (3 weeks saline)	17	17	>3	(A) Weights 3 weeks	Two-way ANOVA	Overall effect of LPS	1, 59	*F* = 23.02	**<0.0001**
	*A* (3 weeks LPS)	16	13			Two-way ANOVA	Overall effect of sex	1, 59	*F* = 4.185	**0.0453**
						Two-way ANOVA	Interaction	1, 59	*F* = 4.312	**0.0422**
						Two-way ANOVA	Effect of LPS in females (Tukey's *post hoc* test)	59	*q* = 6.69	**<0.0001**
						Two-way ANOVA	Effect of LPS in males (Tukey's *post hoc* test)	59	*q* = 2.801	0.2068
	*A* (4 weeks saline)	19	25	>3	(A) Weights 4 weeks	Two-way ANOVA	Overall effect of LPS	1, 79	*F* = 6.013	**0.0164**
	*A* (4 weeks LPS)	23	13			Two-way ANOVA	Overall effect of sex	1, 79	*F* = 19.86	**<0.0001**
						Two-way ANOVA	Interaction	1, 79	*F* = 8.017	**0.0059**
						Two-way ANOVA	Effect of LPS in females (Tukey's *post hoc* test)	79	*q* = 5.041	**0.0034**
						Two-way ANOVA	Effect of LPS in males (Tukey's *post hoc* test)	79	*q* = 0.3994	0.9921
	*A* (6–8 weeks saline)	11	9	>3	(A) Weights 6–8 weeks	Two-way ANOVA	Overall effect of LPS	1, 39	*F* = 13.33	**0.0008**
	*A* (6–8 weeks LPS)	14	9			Two-way ANOVA	Overall effect of sex	1, 39	*F* = 63.97	**<0.0001**
						Two-way ANOVA	Interaction	1, 39	*F* = 0.408	0.5283
						Two-way ANOVA	Effect of LPS in females (Tukey's *post hoc* test)	39	*q* = 3.277	**0.0361**
						Two-way ANOVA	Effect of LPS in males (Tukey's *post hoc* test)	39	*q* = 3.99	0.1115
	*A* 11–14 weeks saline)	11	10	>3	(A) Weights 11–14 weeks	Two-way ANOVA	Overall effect of LPS	1, 38	*F* = 33.45	**<0.0001**
	*A* (11–14 weeks LPS)	13	8			Two-way ANOVA	Overall effect of sex	1, 38	*F* = 82.55	**<0.0001**
						Two-way ANOVA	Interaction	1, 38	*F* = 2.302	0.1375
						Two-way ANOVA	Effect of LPS in females (Tukey's *post hoc* test)	38	*F* = 3.986	**0.0366**
						Two-way ANOVA	Effect of LPS in males (Tukey's *post hoc* test)	38	*F* = 7.898	**<0.0001**
	*A* (14–17 weeks saline)	11	8	>3	(A) Weights 14-17 weeks	Two-way ANOVA	Overall effect of LPS	1, 36	*F* = 1.031	0.3168
	*A* (14–17 weeks LPS )	14	7			Two-way ANOVA	Overall effect of sex	1, 36	*F* = 63.22	**<0.0001**
						Two-way ANOVA	Interaction	1, 36	*F* = 0.004	0.9495
						Two-way ANOVA	Effect of LPS in females (Tukey's *post hoc* test)	36	*q* = 0.8527	0.9305
						Two-way ANOVA	Effect of LPS in males (Tukey's *post hoc* test)	36	*q* = 1.242	0.8161
					(A) Weights all time points	Two-way ANOVA	LPS females vs LPS males	1, 249	*F* = 40.13	**<0.0001**
	*B* (saline)	9	6	>3	(B) Juvenile dyadic interactions	Two-way ANOVA	Effect of LPS (both sexes)	1,28	*F* = 2.189	0.1501
	*B* (LPS)	8	9	>3		Two-way ANOVA	Interaction LPS X sex	1,28	*F* = 7.065	**0.0128**
						Two-way ANOVA	Effect of LPS in females (Tukey's *post hoc* test)	28	*q* = 3.98	**0.0415**
						Two-way ANOVA	Effect of LPS in males (Tukey's *post hoc* test)	28	*q* = 1.229	0.8206
					(C) Open field time in center	RM ANOVA	Effect of condition (treatment + sex)	3, 46	*F* = 2.195	0.3122
	*C* (saline)	8	9	>3		RM ANOVA	Effect of Time	1, 46	*F* = 33.19	**<0.0001**
	*C* (LPS)	10	8	>3		RM ANOVA	Interaction condition × time	3, 46	*F* = 2.195	0.1014
						RM ANOVA	Saline female early vs late (Tukey's *post hoc* test)	46	*t* = 3.898	**0.0013**
****						RM ANOVA	LPS Female early vs late (Tukey's *post hoc* test)	46	*t* = 0.4042	0.9905
****						RM ANOVA	Saline male early vs late (Tukey's *post hoc* test)	46	*t* = 3.644	**0.0027**
****						RM ANOVA	LPS male early vs late (Tukey's *post hoc* test)	46	*t* = 4.194	**0.0005**
Figure 5	*B–D* (saline)	11	9	4	(B) Socialization	Two-tailed *t* test	Effect of LPS in males	23	*t* = 1.856	0.0762
	*B–D* (LPS)	14	8	4		Two-tailed *t* test	Effect of LPS in females	23	*t* = 0.4714	0.6442
					(C-D) Social sniffing habituation	Two-way ANOVA	Effect of LPS in males	1, 44	*F* = 26.99	**0.0073**
	*F–K* (saline)	10	11	5		Two-way ANOVA	Effect of LPS in females	1, 26	*F* = 0.4238	0.5202
	*F–K* (LPS)	13	6	6	(F) DMP latency	RM ANOVA	Effect of sex in saline	1, 114	*F* = 0.359	0.5503
						RM ANOVA	Effect of sex in LPS	1, 102	*F* = 5.832	**0.0175**
					(G) DMP swim speed	RM ANOVA	Effect of sex in saline	1, 114	*F* = 1.259	0.2641
	*M* (saline)	18	18	>3		RM ANOVA	Effect of sex in LPS	1, 102	*F* = 20.85	**<0.0001**

	*M* (LPS)	13	6	>3	(H) DMP thigmotaxia	RM ANOVA	Effect of sex in saline	1, 114	*F* = 4.837	**0.0299**
						RM ANOVA	Effect of sex in LPS	1, 102	*F* = 17.09	**<0.0001**
					(I) DMP previous platform crossings	RM ANOVA	Effect of sex in saline	1, 114	*F* = 0.0008	0.9782
						RM ANOVA	Effect of sex in LPS	1, 102	*F* = 4.324	**0.0401**
					(J) DMP platform annulus entries	RM ANOVA	Effect of sex in saline	1, 114	*F* = 0.0158	0.9001
						RM ANOVA	Effect of sex in LPS	1, 102	*F* = 7.396	**0.0077**
					(L) Marble burying	Two-way ANOVA	Overall effect of LPS	1, 51	*F* = 9.939	**0.0027**
						Two-way ANOVA	Effect of LPS in males (Tukey's *post hoc* test)	51	*q* = 5.248	**0.0028**
						Two-way ANOVA	Effect of LPS in females (Tukey's *post hoc* test)	51	*q* = 1.581	0.6803
Figure 6	All panels and groups	6	6	>3	(B) Cortical thickness	Two-way ANOVA	Effect of LPS	1, 20	*F* = 0.3172	0.5795
						Two-way ANOVA	Effect of sex	1, 20	*F* = 0.3039	0.5875
					(C–G) Density of ant. Satb2 neurons	Two-tailed *t* test	Totals saline male vs LPS male	10	*t* = 2.11	0.0611
						Two-tailed *t* test	Totals saline female vs LPS female	10	*t* = 1.882	0.0893
						Two-way ANOVA	Overall effect of sex in saline	1, 100	*F* = 24.23	**<0.0001**
						Two-way ANOVA	Saline male vs saline female bin 2 Tukey's *post hoc* test)	200	*q* = 4.472	**0.0097**
						Two-way ANOVA	Saline male vs saline female bin 3 Tukey's *post hoc* test)	200	*q* = 4.245	**0.0146**
						Two-way ANOVA	Overall effect of LPS in males	1, 100	*F* = 35.98	**<0.0001**
						Two-way ANOVA	Effect of LPS in males, bin 2 (Tukey's *post hoc* test)	200	*q* = 5.037	**0.0026**
						Two-way ANOVA	Effect of LPS in males, bin 3 (Tukey's *post hoc* test)	200	*q* = 4.764	**0.0050**
						Two-way ANOVA	Effect of LPS in males, bin 4 (Tukey's *post hoc* test)	200	*q* = 3.576	0.0563
						Two-way ANOVA	Overall effect of LPS in females	1, 100	*F* = 26.75	**<0.0001**
						Two-way ANOVA	Effect of LPS in females, bin 2 (Tukey's *post hoc* test)	200	*q* = 1.563	0.6869
						Two-way ANOVA	Effect of LPS in females, bin 3 (Tukey's *post hoc* test)	200	*q* = 1.723	0.6161
						Two-way ANOVA	Effect of LPS in females, bin 4 (Tukey's *post hoc* test)	200	*q* = 1.754	0.6018
						Two-way ANOVA	Baseline effect of sex in saline	1, 100	*F* = 24.23	**<0.0001**
						Two-way ANOVA	Baseline effect of sex, bin 2 (Tukey's *post hoc* test)	200	*q* = 4.472	**0.0097**
						Two-way ANOVA	Baseline effect of sex, bin 3 (Tukey's *post hoc* test)	200	*q* = 4.285	**0.0146**
					(H-L) Density of ant. PV interneurons	Two-tailed *t* test	Totals saline male vs LPS male	10	*t* = 2.536	**0.0295**
						Two-tailed *t* test	Totals saline female vs LPS female	10	*t* = 0.2372	0.8173
						Two-way ANOVA	Overall effect of sex in saline	1, 100	*F* = 24.23	**<0.0001**
						Two-way ANOVA	Saline male vs saline female bin 2 Tukey's *post hoc* test)	200	*q* = 4.988	**0.0029**
						Two-way ANOVA	Saline male vs saline female bin 3 Tukey's *post hoc* test)	200	*q* = 5.635	**0.0005**
						Two-way ANOVA	Saline male vs saline female bin 4 Tukey's *post hoc* test)	200	*q* = 6.795	**<0.0001**
						Two-way ANOVA	Saline male vs saline female bin 5 Tukey's *post hoc* test)	200	*q* = 4.083	**0.0222**
						Two-way ANOVA	Saline male vs saline female bin 6 Tukey's *post hoc* test)	200	*q* = 3.777	**0.0406**
						Two-way ANOVA	Overall effect of LPS in males	1, 100	*F* = 18.79	**<0.0001**
						Two-way ANOVA	Effect of LPS in males, bin 2 (Tukey's *post hoc* test)	200	*q* = 5.097	**0.0022**
						Two-way ANOVA	Effect of LPS in males, bin 3 (Tukey's *post hoc* test)	200	*q* = 4.863	**0.0039**
						Two-way ANOVA	Effect of LPS in males, bin 4 (Tukey's *post hoc* test)	200	*q* = 6.159	**0.0001**
						Two-way ANOVA	Effect of LPS in males, bin 5 (Tukey's *post hoc* test)	200	*q* = 3.341	0.0878
						Two-way ANOVA	Overall effect of LPS in females	1, 100	*F* = 0.1734	0.6730
						Two-way ANOVA	Effect of LPS in females, bin 2 (Tukey's *post hoc* test)	200	*q* = 1.133	0.8539
						Two-way ANOVA	Effect of LPS in females, bin 3 (Tukey's *post hoc* test)	200	*q* = 0.8094	0.9402
						Two-way ANOVA	Effect of LPS in females, bin 4 (Tukey's *post hoc* test)	200	*q* = 0.0665	>0.9999

						Two-way ANOVA	Effect of LPS in females, bin 5 (Tukey's *post hoc* test)	200	*q* = 0.5111	0.9838
						Two-way ANOVA	Baseline effect of sex in saline	1, 100	*F* = 32.89	**<0.0001**
						Two-way ANOVA	Baseline effect of sex, bin 2 (Tukey's *post hoc* test)	200	*q* = 4.988	**0.0356**
						Two-way ANOVA	Baseline effect of sex, bin 3 (Tukey's *post hoc* test)	200	*q* = 5.635	**0.0107**
						Two-way ANOVA	Baseline effect of sex, bin 4 (Tukey's *post hoc* test)	200	*q* = 6.795	**0.0010**
					(M–Q) Density of ant. DAPI cells	One-way ANOVA	Totals all groups	3, 20	*F* = 0.3956	0.7576
						Two-way ANOVA	Overall effect of LPS in males	1, 100	*F* = 9.570	**0.0447**
						Two-way ANOVA	Overall effect of LPS in females	1, 100	*F* = 0.8449	0.3656
						Two-way ANOVA	Baseline effect of sex in saline	1, 100	*F* = 4.282	0.0761
Figure 7	All panels and groups	5	6	>3	(A–E) Density of post. Satb2 neurons	Two-tailed *t* test	Totals saline male vs saline female	9	*t* = 2.426	**0.0382**
						Two-tailed *t* test	Totals saline male vs LPS male	8	*t* = 2.161	0.0626
						Two-tailed *t* test	Totals saline female vs LPS female	10	*t* = 0.1802	0.8606
						Two-way ANOVA	Overall effect of sex in saline	1, 90	*F* = 44.03	**<0.0001**
						Two-way ANOVA	Effect of sex in saline, bin 2 (Tukey's *post hoc* test)	180	*q* = 3.842	**0.0361**
						Two-way ANOVA	Effect of sex in saline, bin 3 (Tukey's *post hoc* test)	180	*q* = 4.245	**0.0161**
						Two-way ANOVA	Effect of sex in saline, bin 5 (Tukey's *post hoc* test)	180	*q* = 5.262	**0.0015**
						Two-way ANOVA	Effect of sex in saline, bin 6 (Tukey's *post hoc* test)	180	*q* = 4.292	**0.0146**
						Two-way ANOVA	Effect of sex in saline, bin 7 (Tukey's *post hoc* test)	180	*q* = 3.868	**0.0343**
						Two-way ANOVA	Effect of sex in saline, bin 8 (Tukey's *post hoc* test)	180	*q* = 3.78	**0.0406**
						Two-way ANOVA	Overall effect of LPS in males	1, 80	*F* = 29.39	**<0.0001**
						Two-way ANOVA	Effect of LPS in males, bin 2 (Tukey's *post hoc* test)	180	*q* = 4.76	**0.0051**
						Two-way ANOVA	Effect of LPS in males, bin #3 (Tukey's *post hoc* test)	180	*q* = 4.113	**0.0211**
						Two-way ANOVA	Effect of LPS in males, bin #5 (Tukey's *post hoc* test)	180	*q* = 4.122	**0.0207**
						Two-way ANOVA	Overall effect of LPS in females	1, 100	*F* = 0.2126	0.6457
						Two-way ANOVA	Baseline effect of sex in saline	1, 100	*F* = 26.250	**<0.0001**
					(F–J) Density of post. PV interneurons	Two-tailed *t* test	Totals saline male vs saline female	9	*t* = 1.392	0.1975
						Two-tailed *t* test	Totals saline male vs LPS male	8	*t* = 2.206	0.0584
						Two-tailed *t* test	Totals saline female vs LPS female	10	*t* = 1.183	0.2643
						Two-way ANOVA	Overall effect of sex in saline	1,90	*F* = 10.18	**0.0020**
						Two-way ANOVA	Overall effect of LPS in males	1, 80	*F* = 0.0399	**<0.0001**
						Two-way ANOVA	Effect of LPS in males, bin 5 (Tukey's *post hoc* test)	180	*q* = 1.276	**0.0259**
						Two-way ANOVA	Overall effect of LPS in females	1, 100	*F* = 6.460	**0.0126**
						Two-way ANOVA	Baseline effect of sex in saline	1, 100	*F* = 5.321	**0.0020**
					(K-O) Density of post. DAPI cells	One-way ANOVA	Totals all groups	3,18	*F* = 0.7322	0.5462
						Two-way ANOVA	Overall effect of sex in saline	1, 90	*F* = 0.1296	0.7197
						Two-way ANOVA	Overall effect of LPS in males	1, 100	*F* = 9.570	**0.0020**
						Two-way ANOVA	Overall effect of LPS in females	1, 100	*F* = 0.8449	0.3602
						Two-way ANOVA	Baseline effect of sex in saline	1, 100	*F* = 4.282	0.7197
Figure 8	Anterior cortex (A–C)	6	6	>3	(A-C) Anterior PV/Satb2 ratio	Two-way ANOVA	Baseline effect of sex in saline	1, 100	*F* = 2.421	0.1229
						Two-way ANOVA	Overall LPS effect in males	1, 100	*F* = 7.731	**0.0065**
						Two-way ANOVA	Effect of LPS in males, bin 3 (Tukey's *post hoc* test)	200	*q* = 3.875	**0.0336**
						Two-way ANOVA	Overall LPS effect in females	1, 100	*F* = 4.54	**0.0356**
						Two-way ANOVA	Effect of LPS in females, bin 3 (Tukey's *post hoc* test)	200	*q* = 0.8335	0.9352
	Posterior cortex (D–F)	5	6	>3	(D-F) Posterior PV/Satb2 ratio	Two-way ANOVA	Baseline effect of sex in saline	1, 100	*F* = 11.36	**0.0011**
						Two-way ANOVA	Effect of sex in saline, bin 7 (Tukey's *post hoc* test)	180	*q* = 4.386	**0.0119**
						Two-way ANOVA	Overall LPS effect in males	1, 80	*F* = 3.456	**0.0298**
						Two-way ANOVA	Overall LPS effect in females	1, 100	*F* = 0.1391	0.7780

Extended Data Figure 1-1	*A, B* (saline 2 h)	Avg. ratios		18	(A) Live/dead ratio	Fisher's exact test	Live/dead ratio Sal vs LPS	NA	NA	**<0.0001**
****	*A, B* (saline 24 h)	Avg. ratios		30	(B) Sex ratio	Fisher's exact test	Sex Ratio Sal vs LPS	NA	NA	0.2001
****	*A, B* (LPS 2 h)	Avg. ratios		13						
****	*A, B* (LPS 24 h)	Avg. ratios		29						
Extended Data Figure 4-1	*A, B* (saline)	11	9	4	(A) Social novelty	Two-way ANOVA	Effect of LPS	1, 38	*F* = 0.0006	0.9807
****	*A, B* (LPS)	14	8	4		Two-way ANOVA	Effect of sex	1, 38	*F* = 0.6419	0.4280
****					(B) Buried food	Two-way ANOVA	Effect of treatment	1, 33	*F* = 1.777	0.1916
****						Two-way ANOVA	Effect of sex	1, 33	*F* = 1.18	0.2852
****	*C* (saline)	8	9	>3	(C) OF total ambulation	Two-way ANOVA	Effect of LPS early	1, 46	*F* = 1.226	0.2739
****	*C* (LPS)	10	8	>3		Two-way ANOVA	Effect of sex early	1, 46	*F* = 3.021	0.0889
****						Two-way ANOVA	Interaction early	1, 46	*F* = 0.2229	0.8820
****						Two-way ANOVA	Effect of LPS late	1, 46	*F* = 1.217	0.2758
****						Two-way ANOVA	Effect of sex late	1, 46	*F* = 1.576	0.2157
****						Two-way ANOVA	Interaction late	1, 46	*F* = 0.2197	0.6415
****						RM ANOVA	Effect of Condition (treatment + sex)	3, 46	*F* = 1.321	0.2790
****						RM ANOVA	Effect of time in saline F (Tukey's *post hoc* test)	46	*t* = 3.543	**0.0037**
****						RM ANOVA	Effect of time in LPS F (Tukey's *post hoc* test)	46	*t* = 1.823	0.2673
****						RM ANOVA	Effect of time in saline M (Tukey's *post hoc* test)	46	*t* = 4.127	**0.0006**
****						RM ANOVA	Effect of time in LPS M (Tukey's *post hoc* test)	46	*t* = 1.79	0.2838

F, Female; M, male; Avg., average; ant., anterior; post., posterior; Sal, saline; df, degrees of freedom. Bold type indicates significance.

**Figure 6.**
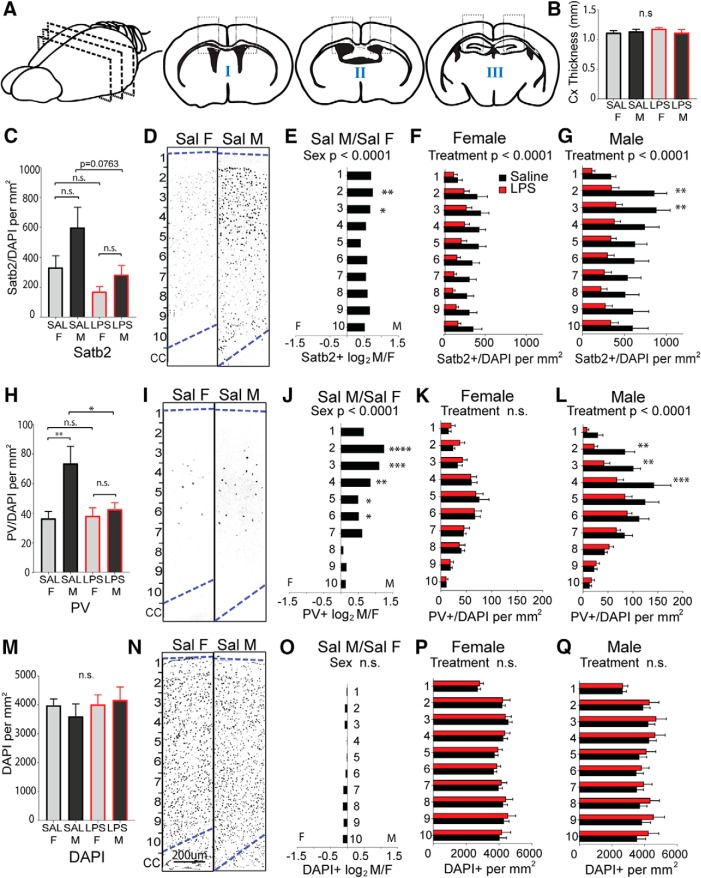
Sex differences in anterior cortical patterning with LPS exposure. Brains were collected from reproductively mature animals that had been exposed to saline or LPS (60 μg/kg) at E12.5. The density of Satb2^+^ cortical projection neurons and PV^+^ interneurons were examined in dorsal anterior cortex, broken down into 10 equal dorsal-to-ventral bins (bin 1 = adjacent to pia, bin 10 = adjacent to corpus callosum boundary). ***A***, Schematic of forebrain regions analyzed for cortical markers: four anterior sections from regions I and II overlying lateral ventricles and the genu of corpus callosum, and two posterior sections from region III overlying rostral hippocampus. ***B***, Total cortical thickness was measured (in millimeters) in the tiled sections from the upper boundary of the corpus callosum to the lower boundary of the pia. ***C***, The density of labeled Satb2^+^ projection neurons was scored for the entire cortical thickness independent of laminar position. ***D***, Representative Satb2 sections from female and male controls. ***E–G***, The density of Satb2^+^ cells in each bin was compared between saline control male and female cortex (***E***), between saline and MIA females (***F***), and saline and MIA males (***G***). ***H***, The density of PV^+^ interneurons was scored for the entire cortical thickness independent of laminar position. ***I***, Representative PV-stained sections from female and male controls. ***J–L***, The density of PV^+^ cells in each bin was compared between saline control male and female cortex (***J***), between saline and MIA females (***K***), and between saline and MIA males (***L***). ***M***, Overall cell density in the cortex was quantified by counting DAPI^+^ nuclei. ***N***, Representative DAPI-stained sections from female and male controls. ***O–Q***, The density of DAPI^+^ cells in each bin was compared between saline control male and female cortices (***O***), between saline and MIA females (***P***), and saline and MIA males (***Q***; mean ± SEM). Saline male (M)/female (F) fold changes depicted as log_2_(M/F). Groups were compared by two-way ANOVA with appropriate *post hoc* tests for individual bins. *p < 0.05, **p < 0.01, ***p < 0.001, ****p < 0.0001, n.s. = not significant.

To determine whether there are sex differences in overall cortical structure or in the two neuronal subtypes most significantly impacted by MIA, sections of cortex from the forebrains of 6- to 8-week-old animals (Fig. 6*A*) were first evaluated for overall cellularity and cortical thickness. There was no measurable effect of treatment or sex on the thickness of the cortex overall (Fig. 6*B*). Anterior sections I and II were next scored for the overall density of Satb2-expressing callosal projection neurons and PV-expressing interneurons. The density of Satb2-labeled neurons was scored for the entire cortical thickness, independent of laminar position (Fig. 6*C*). Counts were then broken down into 10 equally distributed laminar bins and evaluated for layer-selective differences (Fig. 6*D–G*). Interestingly, there was a large and significant baseline sex difference in Satb2^+^ density in the anterior cortex of controls, with female cortex showing lower density than male cortex (Fig. 6*D*). This underlying sex bias in Satb2^+^ neurons was most pronounced in upper layers (Fig. 6*E*). While females exposed to MIA showed an evenly distributed, significant overall decrease in Satb2^+^ neuron density across layers (Fig. 6*F*), the MIA-induced reduction in Satb2^+^ neurons in males was particularly notable in superficial bins 2–3 (approximately corresponding to cortical layers 2/3; Fig. 6*G*).

The density of parvalbumin-labeled neurons was scored for the entire cortical thickness, independent of laminar position (Fig. 6*H*). Counts were then broken down into 10 equally distributed laminar bins and evaluated for layer-selective differences (Fig. 6*I–L*). PV^+^ cortical interneurons showed a strong baseline sex bias toward a higher density in upper layers of the control male cortex (Fig. 6*I*,*J*). PV^+^ density was not affected by MIA in females (Fig. 6*K*) and showed an MIA-induced decrease in males only, an effect that was most pronounced in the upper cortical lamina (Fig. 6*L*).

**Figure 7.**
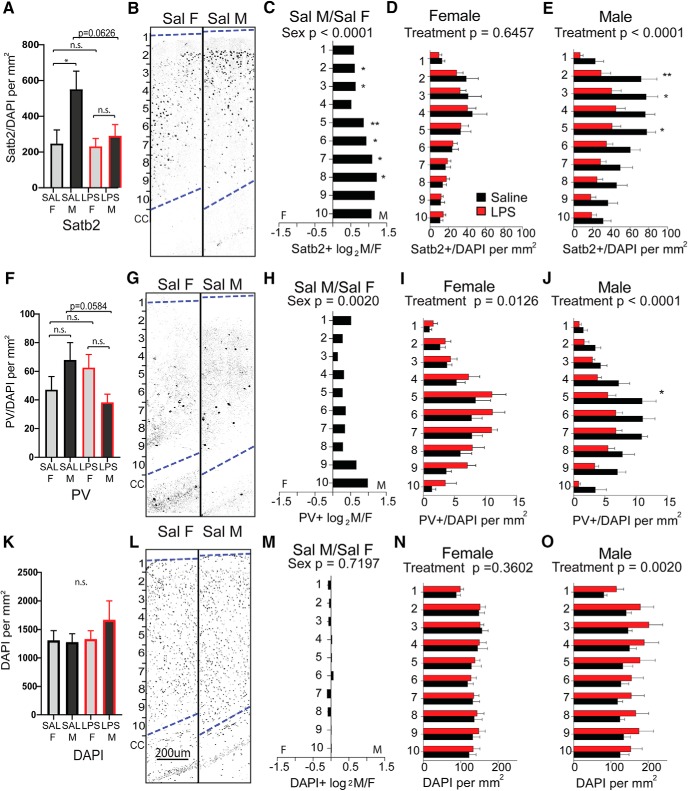
Sex differences in posterior cortical patterning with LPS exposure. Brains were collected from reproductively mature animals that had been exposed to saline or LPS (60 μg/kg) at E12.5. The density of Satb2^+^ cortical projection neurons and PV^+^ interneurons were examined in two sections of dorsal posterior cortex (region III), broken down into 10 equal dorsal-to-ventral bins (bin 1 = adjacent to pia, bin 10 = adjacent to corpus callosum boundary). ***A***, The density of labeled Satb2^+^ projection neurons was scored for the entire cortical thickness independent of laminar position. ***B***, Representative Satb2 sections from male and female controls. ***C–E***, The density of Satb2^+^ cells in each bin was compared between saline control male and female cortex (***C***), between saline and MIA males (***D***), and between saline and MIA females (***E***). ***F***, The density of labeled PV^+^ interneurons was scored for the entire cortical thickness independent of laminar position. ***G***, Representative PV-stained sections from male and female controls. ***H–J***, The density of PV^+^ cells in each bin was compared between saline control male and female cortex (***H***), between saline and MIA males (***I***), and between saline and MIA females (***J***). ***K***, Overall cell density in the cortex was quantified by counting DAPI^+^ nuclei. ***L***, Representative DAPI-stained sections from male and female controls. ***M–O***, The density of DAPI^+^ cells in each bin was compared between saline control male and female cortices (***M***), between saline and MIA males (***N***), and between saline and MIA females (***O***). Groups were compared by two-way ANOVA with appropriate *post hoc* tests for individual bins. *p < 0.05, **p < 0.01, n.s. = not significant.

In more posterior areas of neocortex overlying the hippocampus (Fig. 6*A*, section III), the baseline sex difference in Satb2^+^ density was also observable (Fig. 7*A–C*). However, in this region Satb2^+^ density was unaffected by MIA in females (Fig. 7*D*) and was decreased in males only (Fig. 7*E*). The baseline sex difference observed in PV^+^ in anterior cortex was observable in posterior cortex as well (Fig. 7*F–H*). Notably, while PV^+^ density was decreased by MIA in posterior male cortex in a manner resembling the effect in anterior cortex, females exhibited an effect in the opposite direction, with PV^+^ cells significantly more densely distributed in posterior cortex (Fig. 7*I*,*J*). Although overall DAPI^+^ cellularity was not different by sex (Figs. 6*M–O*, 7*K–M*), in both the anterior and posterior sections of cortex, MIA caused an overall increase in DAPI^+^ cell density in males only (Figs. 6*P*,*Q*, 7*N*,*O*).

**Figure 8.**
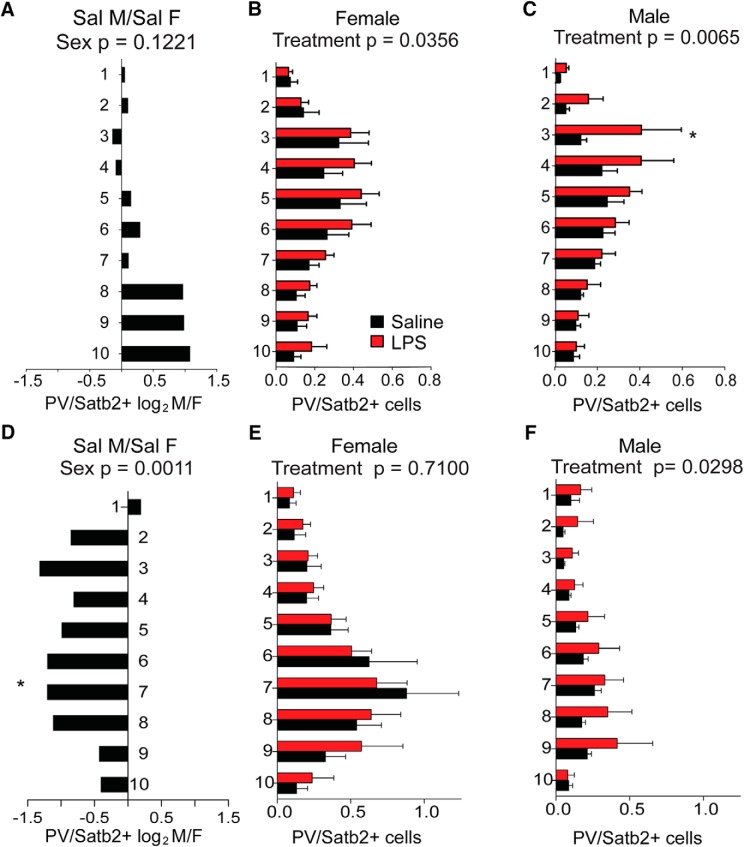
PV/Satb2 ratios in anterior and posterior adult cortices. ***A–C***, The ratio of PV^+^ interneurons to Satb2^+^ projection neurons was calculated in anterior cortex (sections I–III) and compared between saline control male and female cortices (***A***), between saline and MIA females (***B***), and between saline and MIA males (***C***). ***D–F***, The ratio of PV^+^ interneurons to Satb2^+^ projection neurons was calculated in posterior cortex (section IV) and compared between saline control male and female cortices (***D***), between saline and MIA females (***E***), and between saline and MIA males (***F***). Mean ± SEM. PV/Satb2 ratio groups compared by two-way ANOVA with appropriate *post hoc* tests for individual bins. *p < 0.05, n.s. = not significant.

To determine how compounded changes in the density of neuronal subpopulations may impact overall cortical organization, we examined the ratio of PV^+^ to Satb2^+^ cells in both anterior and posterior sections of cortex. While there were large baseline sex differences in the density of both cell types in anterior cortex, the ratio of PV/Satb2 density in this region is not significantly different between the sexes in the control condition (Fig. 8*A*). The ratio of PV/Satb2 was slightly increased by MIA in female cortex, with no particular layer specifically affected (Fig. 8*B*), while in males MIA significantly increased the ratio of PV/Satb2 throughout the cortex, particularly in layer II/III (Fig. 8*C*). In posterior cortex, there was a baseline sex difference in PV/Satb2 ratio with significantly higher PV to Satb2 density in control females (Fig. 8*D*). In posterior sections, the PV/Satb2 ratio was unaffected by MIA in females (Fig. 8*E*) and was significantly increased by MIA in males, with no particular layer specifically affected (Fig. 8*F*).

## Discussion

Maternal infections and other immune events in early gestation are associated with increased risk of neurodevelopmental disorders such as ASD (Meyer et al., 2006, 2007). Male sex has also been described as a risk factor for neurodevelopmental disorders (Werling and Geschwind, 2013) and complications during gestation (Eriksson et al., 2010; Sandman et al., 2013; Broere-Brown, 2017). This male bias is well recognized; however, females are also affected in large numbers, and there is little empirical data directly contrasting outcomes in separate male and female cohorts (Beery and Zucker 2011; Coiro and Pollak 2019). The predominant assumption is that the disease mechanisms are shared by both sexes, but that the female sex is “protective,” simply attenuating the degree of pathology. Here we find that fetal sex interacts with MIA to generate unique sex-specific effects in offspring.

At the level of the placenta, males showed reduced placental growth and more severe MIA-induced necrosis, with the lowest tested dose of LPS causing damage in males but not in females (Fig. 1). During this acute phase of the maternal immune response, umbilical blood flow and fetal heart rate were decreased (Fig. 2*F*,*G*) and the resulting hypoxia in the developing cortex was more severe in males (Fig. 3*A*,*B*). This was accompanied by a male-biased reduction in M-phase cells in the cortical ventricular zone, while females showed either an absence of effect or greatly attenuated acute effects (Fig. 3*C*,*D*). This constellation of effects suggests that males are more vulnerable to the acute effects of maternal inflammation on both placental function and fetal brain development.

Male rat placenta shows more severe effects of bacterial infection in the placental labyrinth, which was associated with more pronounced autism-like behaviors in adult offspring (Allard et al., 2017). However, in contrast to a direct bacterial infection model (Makinson et al., 2017), it is highly unlikely that LPS acts directly on the placenta in our present model and instead results from the impact of MIA-induced circulating maternal cytokines. Our earlier work showed that deletion of the LPS receptor TLR4 in fetal tissues, including placenta, was not protective, indicating that LPS did not cause placental bleeding and tissue necrosis by acting directly on placenta and required maternal TLR4. We also demonstrated that maternal TNFα acting on the fetal TNFR1 in the placenta is necessary for the placental damage and fetal loss observed after LPS treatment (Carpentier et al., 2011). We show here that TNFα is significantly more abundant in male placenta 2 h after MIA (Fig. 2*A*) and that there was an overall trend for cytokine abundance to be higher in male placenta relative to female placenta (Fig. 2*B–E*).

The most abundant cytokine induced by MIA in placenta was CXCL1, which was significantly elevated in male versus female placentas (Fig. 2*E*). The production of CXCL1 is elicited by TNFα, and it is possible that the placental output of CXCL1 in response to maternal TNFα represents a male-biased amplification of the inflammatory signaling cascade within the placenta. Males also show a higher abundance of proinflammatory cytokines in placenta tissues after maternal exposure to chronic variable stress, suggesting a convergence in sex-specific vulnerability between immune and psychosocial stress-mediated prenatal insults (Bronson and Bale, 2014; Nugent et al., 2018). It is unknown whether similar sex differences in inflammatory response exist at mid-gestation in human placenta; however, when cultured trophoblasts from healthy human term placenta were treated with LPS, male cells selectively produced more TNFα. TNF and nuclear factor-κB signaling networks have been identified as hubs of sex-divergent gene expression in human placental cells (Cvitic et al., 2013), indicating that sex differences in placental inflammatory signaling are likely to be conserved between species.

In contrast to the placenta, the female fetal brain was unique in that CXCL10, a chemokine that has been identified as an inflammatory contributor to anxiety in mice (Blank et al., 2016; Davis et al., 2017), was selectively elevated at 2 h after MIA (Fig. 3*H*). It is interesting to note that the number and relative abundance of cytokines that are elevated in maternal serum 2 h after LPS treatment is higher than the number of cytokines elevated in fetal placenta (Carpentier et al., 2011), which is itself higher than observed in fetal brain (Fig. 3*G*), suggesting that despite some propagation of the inflammatory response from maternal to fetal tissue, the fetal brain is still substantially buffered from the direct inflammatory impact of MIA, perhaps with sex-selective permeability.

Several consequences of the *in utero* disruption are observed in postnatal animals. MIA females gained weight more slowly than control females from postnatal week 3 to 14, while male body weight gain was normal overall with a transient delay in adult growth only (Fig. 4*A*). This observation aligns with the hypothesis that males in polygynous, sexually dimorphic species will preserve somatic growth potential in response to prenatal adversity, while females more readily sacrifice body growth to improve survival and, perhaps, to protect brain development (Clifton, 2010).

The behavioral consequences of MIA include a female-specific increase in the initiation of dyadic interactions at P21, suggesting an increase in tendency to interact socially during this period (Fig. 4*C*). In adult, we note a male-selective reduction in adult social interaction and impaired spatial learning, and an increase in repetitive behavior (Fig. 5). These behaviors are considered to be somewhat analogous to human social aversion, learning disability, and behavioral stereotypies, which are common features of ASDs (Baron-Cohen et al., 2011; Werling and Geschwind, 2013). These reciprocal social effects in females and males should be noted as an example that male and female outcomes can be mutually exclusive and even opposite. As adults, MIA females showed sex-specific habituation deficits in the open field test and were unique in their prolonged avoidance of exploration in the center of the arena during the latter phase of the test. This tendency to remain at the extreme periphery of the arena may indicate a female-selective effect of MIA on anxiety-related behavior (Fig. 4*D*; Treit and Fundytus, 1988). Interestingly, human anxiety disorders show an opposite sex bias compared with neurodevelopmental disorders, with two females affected for every male (Altemus et al., 2014).

Cortical development is precisely coordinated, and the timing of the terminal cell division is important to determine the fate and permanent location of that neuron (Fame et al., 2011). Our earlier work has also shown that LPS-induced MIA has pronounced effects on the abundance of two neuronal subtypes in the adult cortex, Satb2^+^ callosal projection neurons and parvalbumin^+^ cortical interneurons. PV interneurons play a critical role in modulating local communication in the cortical lamina and can strongly influence both corticocortical and subcortical projection neuron functions. Examining these cells in both male and female cortices, we discovered a pronounced baseline sex difference in the abundance of Satb2^+^ callosal projection neurons and PV^+^ interneurons, with normal males exhibiting significantly higher densities in all regions examined (Figs. 6, 7).

It is intriguing to note that many of the effects of MIA in males could be described as more prominently acting on features that are naturally dimorphic. For example, a male placenta normally grows at a higher rate between E12.5 and E13.5 (Fig. 1), and with exposure to MIA its growth trajectory is decreased and begins to resemble that of normal females, while the female placenta shows no growth impairment with MIA. It has been demonstrated that a placenta in the developing phase (Knox and Baker, 2008) during early pregnancy is more vulnerable to LPS-induced damage (Carpentier et al., 2011). It is possible that increased metabolic burden of male growth relative to female growth at the time of challenge puts males at greater risk for placental pathology, and potentially also for downstream fetal hypoxia. The effects of this energetic crisis on male brain may then exceed a resilience threshold, selectively impacting the mechanisms of corticogenesis in males, with female brains exhibiting greater robustness to the same disruption due to the comparatively lower metabolic demands of placental growth and brain development.

In another example of pre-existing sexual dimorphism affecting outcome, the elevated baseline density of Satb2^+^ and PV^+^ neurons in male cortex is substantially reduced by MIA throughout all regions examined, whereas female cortex begins at a comparatively lower density and shows little to no reduction of these cell types (Figs. 6, 7). It is possible that the dampening of the mitotic rate in male ventricular zone contributes to the pronounced and specific depletion of Satb2^+^ cells in the adult male cortex, as this population normally begins to appear in cortical plate in the window following the E12.5 insult (Alcamo et al., 2008; Britanova et al., 2008; Leone et al., 2008; Kwan et al., 2012; Greig et al., 2013). It has been demonstrated that radially migrating excitatory projection neurons in the developing cortical plate guide the transverse migration of interneurons from the ganglionic eminences (Lodato et al., 2011), suggesting that the depleted population of Satb2^+^ cells in the male cortex may recruit fewer PV^+^ interneurons into the same layers of cortex. If higher densities of Satb2 and PV neurons are required to achieve normal cortical cytoarchitecture in males, then this requirement for higher cell production in the mid-gestational cortical plate may present a unique point of male vulnerability that interacts with vulnerabilities in other tissues in a complex synergistic manner. While it is still unclear precisely how these changes in cell population densities in cortex manifest at the circuit level, we have previously demonstrated that the MIA-induced decrease in Satb2^+^ cell density in cortex is accompanied by the loss of commissural Satb2^+^ projections in the corpus callosum (Carpentier et al., 2013), which may contribute to an “underconnected” cortex due to a loss of long-range corticocortical projections. It has been proposed that the brains of people with ASD exhibit cortical underconnectivity (Geschwind and Levitt, 2007; Just et al. (2007), as well as decreased callosal volumes (Frazier and Hardan, 2009; Wegiel et al. (2018).

Notably, while MIA dramatically decreased PV cell density in males in both anterior and posterior cortex (Figs. 6*L*, 7*J* ), PV density was unaffected in anterior female cortex (Fig. 6*K*) and significantly increased in posterior female cortex (Fig. 7*I*), indicating that neurodevelopmental outcomes in females are not simply an attenuated version of male outcomes, but can differ in both region and direction of change. The covarying relationship between PV and Satb2 neuron densities observed in males does not hold true for female brains exposed to MIA, in which anterior PV density is unaffected while Satb2 density decreases and posterior PV density increases while Satb2 density is unchanged (Figs. 7, 8). This decoupling of PV and Satb2 neuron population density changes is unique to females, suggesting that developmental relationships between neuronal subtypes in fetal cortex may have sex-specific features. Given that PV^+^ interneurons are thought to set the excitatory/inhibitory balance in cortex, decreasing the signal-to-noise ratio to drive appropriate behavioral output (Ferguson and Gao, 2018), it is possible that the observed sex-selective changes in PV^+^ neuron organization and PV^+^/Satb2^+^ balance contribute to the distinct behavioral impacts of MIA on male and female offspring.

**Figure 9.**
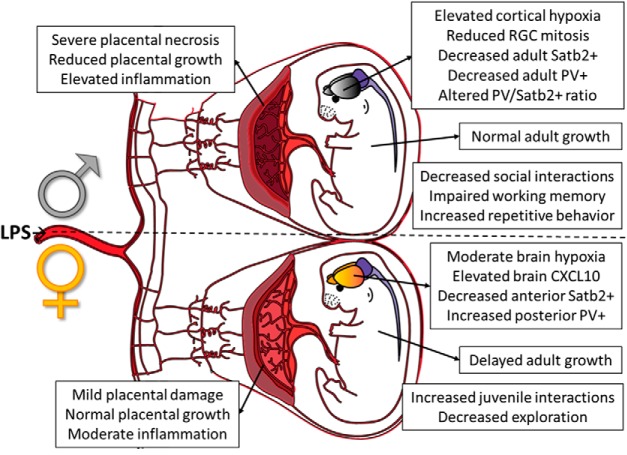
Graphical summary of sex-divergent developmental consequences resulting from LPS-induced maternal immune activation.

The data presented here provide a conceptual outline for how sex differences in response to the same acute inflammatory insult can be propagated across the fetal–maternal interface to the developing brain, producing sex-dependent neuroanatomical and behavioral effects that persist to adulthood (Fig. 9, graphical summary). We find that females are not simply protected from the consequences of the inflammatory event but experience their own unique developmental response to maternal inflammatory signaling. These findings highlight the role that baseline sex differences play in defining risk mechanisms and outcomes, and reinforce the value of consistently examining the mechanisms that underlie health and disease states in both sexes.

## Synthesis

Reviewing Editor: Cheryl Sisk, Michigan State University Decisions are customarily a result of the Reviewing Editor and the peer reviewers coming together and discussing their recommendations until a consensus is reached. When revisions are invited, a fact-based synthesis statement explaining their decision and outlining what is needed to prepare a revision will be listed below. The following reviewer(s) agreed to reveal their identity: NONE. Note: If this manuscript was transferred from JNeurosci and a decision was made to accept the manuscript without peer review, a brief statement to this effect will instead be what is listed below.

Authors have satisfactorily responded to the previous review.

## References

[B1] Alcamo EA, Chirivella L, Dautzenberg M, Dobreva G, Fariñas I, Grosschedl R, McConnell SK (2008) Satb2 regulates callosal projection neuron identity in the developing cerebral cortex. Neuron 57:364–377. 10.1016/j.neuron.2007.12.012 18255030

[B2] Allard MJ, Bergeron JD, Baharnoori M, Srivastava LK, Fortier LC, Poyart C, Sébire G (2017) A sexually dichotomous, autistic-like phenotype is induced by group B streptococcus maternofetal immune activation. Autism Res 10:233–245. 10.1002/aur.1647 27220806

[B3] Altemus M, Sarvaiya N, Neill Epperson C (2014) Sex differences in anxiety and depression clinical perspectives. Front Neuroendocrinol 35:320–330. 10.1016/j.yfrne.2014.05.004 24887405 PMC4890708

[B4] Atladóttir HO, Thorsen P, Østergaard L, Schendel DE, Lemcke S, Abdallah M, Parner ET (2010) Maternal infection requiring hospitalization during pregnancy and autism spectrum disorders. J Autism Dev Disord 40:1423–1430. 10.1007/s10803-010-1006-y 20414802

[B5] Baron-Cohen S, Lombardo MV, Auyeung B, Ashwin E, Chakrabarti B, Knickmeyer R (2011) Why are autism spectrum conditions more prevalent in males? PLoS Biol 9:e1001081. 10.1371/journal.pbio.1001081 21695109 PMC3114757

[B6] Beery AK, Zucker I (2011) Sex bias in neuroscience and biomedical research. Neurosci Biobehav Rev 35:565–572. 10.1016/j.neubiorev.2010.07.002 20620164 PMC3008499

[B7] Beversdorf DQ, Manning SE, Hillier A, Anderson SL, Nordgren RE, Walters SE, Nagaraja HN, Cooley WC, Gaelic SE, Bauman ML (2005) Timing of prenatal stressors and autism. J Autism Dev Disord 35:471–478. 10.1007/s10803-005-5037-8 16134032

[B8] Blank T, Detje CN, Spieß A, Hagemeyer N, Brendecke SM, Wolfart J, Staszewski O, Zöller T, Papageorgiou I, Schneider J, Paricio-Montesinos R, Eisel UL, Manahan-Vaughan D, Jansen S, Lienenklaus S, Lu B, Imai Y, Müller M, Goelz SE, Baker DP, et al (2016) Brain endothelial- and epithelial-specific interferon receptor chain 1 drives virus-induced sickness behavior and cognitive impairment. Immunity 44:901–912. 10.1016/j.immuni.2016.04.005 27096319

[B9] Bozdagi O, Sakurai T, Papapetrou D, Wang X, Dickstein DL, Takahashi N, Kajiwara Y, Yang M, Katz AM, Scattoni ML, Harris MJ, Saxena R, Silverman JL, Crawley JN, Zhou Q, Hof PR, Buxbaum JD (2010) Haploinsufficiency of the autism-associated Shank3 gene leads to deficits in synaptic function, social interaction, and social communication. Mol Autism 1:15. 10.1186/2040-2392-1-15 21167025 PMC3019144

[B10] Britanova O, de Juan Romero C, Cheung A, Kwan KY, Schwark M, Gyorgy A, Vogel T, Akopov S, Mitkovski M, Agoston D, Sestan N, Molnár Z, Tarabykin V (2008) Satb2 is a postmitotic determinant for upper-layer neuron specification in the neocortex. Neuron 57:378–392. 10.1016/j.neuron.2007.12.028 18255031

[B11] Broere-Brown Z (2017) Fetal sex dependency in pregnancy; fetal and maternal outcomes, the Generation R Study. PhD thesis, Erasmus University Rotterdam.

[B12] Bronson SL, Bale TL (2014) Prenatal stress-induced increases in placental inflammation and offspring hyperactivity are male-specific and ameliorated by maternal anti-inflammatory treatment. Endocrinology 155:2635–2646. 10.1210/en.2014-1040 24797632 PMC4060181

[B13] Brown AS, Schaefer CA, Wyatt RJ, Goetz R, Begg MD, Gorman JM, Susser ES (2000) Maternal exposure to respiratory infections and adult schizophrenia spectrum disorders: a prospective birth cohort study. Schizophr Bull 26:287–295. 10.1093/oxfordjournals.schbul.a033453 10885631

[B14] Carpentier PA, Dingman AL, Palmer TD (2011) Placental TNF-α signaling in illness-induced complications of pregnancy. Am J Pathol 178:2802–2810. 10.1016/j.ajpath.2011.02.042 21641402 PMC3124299

[B15] Carpentier PA, Haditsch U, Braun AE, Cantu AV, Moon HM, Price RO, Anderson MP, Saravanapandian V, Ismail K, Rivera M, Weimann JM, Palmer TD (2013) Stereotypical alterations in cortical patterning are associated with maternal illness-induced placental dysfunction. J Neurosci 33:16874–16888. 10.1523/JNEUROSCI.4654-12.2013 24155294 PMC3807021

[B17] Challis J, Newnham J, Petraglia F, Yeganegi M, Bocking A (2013) Fetal sex and preterm birth. Placenta 34:95–99. 10.1016/j.placenta.2012.11.007 23261268

[B18] Clifton VL (2010) Review: sex and the human placenta: mediating differential strategies of fetal growth and survival. Placenta 31 [Suppl]:S33–S39. 10.1016/j.placenta.2009.11.010 20004469

[B19] Coiro P, Pollak DD (2019) Sex and gender bias in the experimental neurosciences: the case of the maternal immune activation model. Transl Psychiatry 9:90. 10.1038/s41398-019-0423-8 30765690 PMC6375995

[B20] Crawley JN (2007) Mouse behavioral assays relevant to the symptoms of autism. Brain Pathol 17:448–459. 10.1111/j.1750-3639.2007.00096.x 17919130 PMC8095652

[B21] Croen LA, Grether JK, Yoshida CK, Odouli R, Van de Water J (2005) Maternal autoimmune diseases, asthma and allergies, and childhood autism spectrum disorders: a case-control study. Arch Pediatr Adolesc Med 159:151–157. 10.1001/archpedi.159.2.151 15699309

[B22] Custódio CS, Mello BSF, Filho A, de Carvalho Lima CN, Cordeiro RC, Miyajima F, Réus GZ, Vasconcelos SMM, Barichello T, Quevedo J, de Oliveira AC, de Lucena DF, Macedo DS (2018) Neonatal immune challenge with lipopolysaccharide triggers long-lasting sex- and age-related behavioral and immune/neurotrophic alterations in mice: relevance to autism spectrum disorders. Mol Neurobiol 55:3775–3788. 10.1007/s12035-017-0616-1 28536974

[B23] Cvitic S, Longtine MS, Hackl H, Wagner K, Nelson MD, Desoye G, Hiden U (2013) The human placental sexome differs between trophoblast epithelium and villous vessel endothelium. PLoS One 8:e79233. 10.1016/j.placenta.2013.06.209 24205377 PMC3812163

[B24] Davis RL, Stevens CW, Curtis JT (2017) The opioid antagonist, β-funaltrexamine, inhibits lipopolysaccharide-induced neuroinflammation and reduces sickness behavior in mice. Physiol Behav 173:52–60. 10.1016/j.physbeh.2017.01.037 28130086

[B25] Eloundou SN, Lee J, Wu D, Lei J, Feller MC, Ozen M, Zhu Y, Hwang M, Jia B, Xie H, Clemens JL, McLane MW, AlSaggaf S, Nair N, Wills-Karp M, Wang X, Graham EM, Baschat A, Burd I (2019) Placental malperfusion in response to intrauterine inflammation and its connection to fetal sequelae. PLoS One 14:e0214951. 10.1371/journal.pone.0214951 30943260 PMC6447225

[B26] Eriksson JG, Kajantie E, Osmond C, Thornburg K, Barker DJ (2010) Boys live dangerously in the womb. Am J Hum Biol 22:330–335. 10.1002/ajhb.20995 19844898 PMC3923652

[B27] Fame RM, MacDonald JL, Macklis JD (2011) Development, specification, and diversity of callosal projection neurons. Trends Neurosci 34:41–50. 10.1016/j.tins.2010.10.002 21129791 PMC3053014

[B28] Fatemi SH, Folsom TD, Rooney RJ, Mori S, Kornfield TE, Reutiman TJ, Kneeland RE, Liesch SB, Hua K, Hsu J, Patel DH (2012) The viral theory of schizophrenia revisited: abnormal placental gene expression and structural changes with lack of evidence for H1N1 viral presence in placentae of infected mice or brains of exposed offspring. Neuropharmacology 62:1290–1298. 10.1016/j.neuropharm.2011.01.011 21277874 PMC3156896

[B29] Ferguson BR, Gao WJ (2018) PV interneurons: critical regulators of e/i balance for prefrontal cortex-dependent behavior and psychiatric disorders. Front Neural Circuits 12:37. 29867371 10.3389/fncir.2018.00037 PMC5964203

[B30] Frazier TW, Hardan AY (2009) A meta-analysis of the corpus callosum in autism. Biol Psychiatry 66:935–941. 10.1016/j.biopsych.2009.07.022 19748080 PMC2783565

[B31] Froehlich-Santino W, Londono Tobon A, Cleveland S, Torres A, Phillips J, Cohen B, Torigoe T, Miller J, Fedele A, Collins J, Smith K, Lotspeich L, Croen LA, Ozonoff S, Lajonchere C, Grether JK, O'Hara R, Hallmayer J (2014) Prenatal and perinatal risk factors in a twin study of autism spectrum disorders. J Psychiatr Res 54:100–108. 10.1016/j.jpsychires.2014.03.019 24726638 PMC4072527

[B32] Gendron RL, Nestel FP, Lapp WS, Baines MG (1990) Lipopolysaccharide-induced fetal resorption in mice is associated with the intrauterine production of tumour necrosis factor-alpha. J Reprod Fertil 90:395–402. 10.1530/jrf.0.0900395 2250238

[B33] Geschwind DH, Levitt P (2007) Autism spectrum disorders: developmental disconnection syndromes. Curr Opin Neurobiol 17:103–111. 10.1016/j.conb.2007.01.009 17275283

[B34] Goines PE, Croen LA, Braunschweig D, Yoshida CK, Grether J, Hansen R, Kharrazi M, Ashwood P, Van de Water J (2011) Increased midgestational IFN-g, IL-4 and IL-5 in women bearing a child with autism: a case-control study. Mol Autism 2:13. 10.1186/2040-2392-2-13 21810230 PMC3170586

[B35] Golan HM, Lev V, Hallak M, Sorokin Y, Huleihel M (2005) Specific neurodevelopmental damage in mice offspring following maternal inflammation during pregnancy. Neuropharmacology 48:903–917. 10.1016/j.neuropharm.2004.12.023 15829260

[B36] Greig LC, Woodworth MB, Galazo MJ, Padmanabhan H, Macklis JD (2013) Molecular logic of neocortical projection neuron specification, development and diversity. Nat Rev Neurosci 14:755–769. 10.1038/nrn3586 24105342 PMC3876965

[B78] Grove J, Ripke S, Als TD, Mattheisen M, Walters RK, Won H, Pallesen J, Agerbo E, Andreassen OA, Anney R, Awashti S, Belliveau R, Bettella F, Buxbaum JD, Bybjerg-Grauholm J, Bækvad-Hansen M, Cerrato F, Chambert K, Christensen JH, Churchhouse C, et al (2019) Identification of common genetic risk variants for autism spectrum disorder. Nat Genet 51:431–444. 10.1038/s41588-019-0344-8 30804558 PMC6454898

[B37] Haditsch U, Leone DP, Farinelli M, Chrostek-Grashoff A, Brakebusch C, Mansuy IM, McConnell SK, Palmer TD (2009) A central role for the small GTPase Rac1 in hippocampal plasticity and spatial learning and memory. Mol Cell Neurosci 41:409–419. 10.1016/j.mcn.2009.04.005 19394428 PMC2705331

[B38] Haida O, Al Sagheer T, Balbous A, Francheteau M, Matas E, Soria F, Fernagut PO, Jaber M (2019) Sex-dependent behavioral deficits and neuropathology in a maternal immune activation model of autism. Transl Psychiatry 9:124. 10.1038/s41398-019-0457-y 30923308 PMC6438965

[B39] Hallmayer J, Cleveland S, Torres A, Phillips J, Cohen B, Torigoe T, Miller J, Fedele A, Collins J, Smith K, Lotspeich L, Croen LA, Ozonoff S, Lajonchere C, Grether JK, Risch N (2011) Genetic heritability and shared environmental factors among twin pairs with autism. Arch Gen Psychiatry 68:1095–1102. 10.1001/archgenpsychiatry.2011.76 21727249 PMC4440679

[B41] Jacquemont S, Coe BP, Hersch M, Duyzend MH, Krumm N, Bergmann S, Beckmann JS, Rosenfeld JA, Eichler EE (2014) A higher mutational burden in females supports a “female protective model” in neurodevelopmental disorders. Am J Hum Genet 94:415–425. 10.1016/j.ajhg.2014.02.001 24581740 PMC3951938

[B42] Just MA, Cherkassky VL, Keller TA, Kana RK, Minshew NJ (2007) Functional and anatomical cortical underconnectivity in autism: evidence from an FMRI study of an executive function task and corpus callosum morphometry. Cereb Cortex 17:951–961. 10.1093/cercor/bhl006 16772313 PMC4500121

[B43] Knox K, Baker JC (2008) Genomic evolution of the placenta using co-option and duplication and divergence. Genome Res 18:695–705. 18340042 10.1101/gr.071407.107 PMC2336813

[B44] Kwan KY, Sestan N, Anton ES (2012) Transcriptional co-regulation of neuronal migration and laminar identity in the neocortex. Development 139:1535–1546. 10.1242/dev.069963 22492350 PMC3317962

[B45] Lee BK, Magnusson C, Gardner RM, Blomström Å, Newschaffer CJ, Burstyn I, Karlsson H, Dalman C (2015) Maternal hospitalization with infection during pregnancy and risk of autism spectrum disorders. Brain Behav Immun 44:100–105. 10.1016/j.bbi.2014.09.001 25218900 PMC4418173

[B46] Leone DP, Srinivasan K, Chen B, Alcamo E, McConnell SK (2008) The determination of projection neuron identity in the developing cerebral cortex. Curr Opin Neurobiol 18:28–35. 10.1016/j.conb.2008.05.006 18508260 PMC2483251

[B47] Lodato S, Rouaux C, Quast KB, Jantrachotechatchawan C, Studer M, Hensch TK, Arlotta P (2011) Excitatory projection neuron subtypes control the distribution of local inhibitory interneurons in the cerebral cortex. Neuron 69:763–779. 10.1016/j.neuron.2011.01.015 21338885 PMC3061282

[B48] Makinson R, Lloyd K, Rayasam A, McKee S, Brown A, Barila G, Grissom N, George R, Marini M, Fabry Z, Elovitz M, Reyes TM (2017) Intrauterine inflammation induces sex-specific effects on neuroinflammation, white matter, and behavior. Brain Behav Immun 66:277–288. 10.1016/j.bbi.2017.07.016 28739513 PMC6916731

[B49] Mandy W, Chilvers R, Chowdhury U, Salter G, Seigal A, Skuse D (2012) Sex differences in autism spectrum disorder: evidence from a large sample of children and adolescents. J Autism Dev Disord 42:1304–1313. 10.1007/s10803-011-1356-0 21947663

[B50] Meyer U, Nyffeler M, Engler A, Urwyler A, Schedlowski M, Knuesel I, Yee BK, Feldon J (2006) The time of prenatal immune challenge determines the specificity of inflammation-mediated brain and behavioral pathology. J Neurosci 26:4752–4762. 10.1523/JNEUROSCI.0099-06.2006 16672647 PMC6674174

[B51] Meyer U, Yee BK, Feldon J (2007) The neurodevelopmental impact of prenatal infections at different times of pregnancy: the earlier the worse? Neuroscientist 13:241–256. 10.1177/1073858406296401 17519367

[B52] Mitra I, Tsang K, Ladd-Acosta C, Croen LA, Aldinger KA, Hendren RL, Traglia M, Lavillaureix A, Zaitlen N, Oldham MC, Levitt P, Nelson S, Amaral DG, Hertz-Picciotto I, Fallin MD, Weiss LA (2016) Pleiotropic mechanisms indicated for sex differences in autism. PLoS Genet 12:e1006425 10.1371/journal.pgen.1006425 27846226 PMC5147776

[B53] Molyneaux BJ, Arlotta P, Menezes JR, Macklis JD (2007) Neuronal subtype specification in the cerebral cortex. Nat Rev Neurosci 8:427–437. 10.1038/nrn2151 17514196

[B54] Mueller FS, Richetto J, Hayes LN, Zambon A, Pollak DD, Sawa A, Meyer U, Weber-Stadlbauer U (2019) Influence of poly(I:C) variability on thermoregulation, immune responses and pregnancy outcomes in mouse models of maternal immune activation. Brain Behav Immun 80:406–418. 30980948 10.1016/j.bbi.2019.04.019

[B55] Nugent BM, O'Donnell CM, Epperson CN, Bale TL (2018) Placental H3K27me3 establishes female resilience to prenatal insults. Nat Commun 9:2555. 10.1038/s41467-018-04992-1 29967448 PMC6028627

[B56] Ornoy A, Weinstein-Fudim L, Ergaz Z (2016) Genetic syndromes, maternal diseases, and antenatal factors associated with autism spectrum disorders (ASD). Front Neurosci 10:316. 27458336 10.3389/fnins.2016.00316 PMC4933715

[B57] Patel TP, Gullotti DM, Hernandez P, O'Brien WT, Capehart BP, Morrison B 3rd, Bass C, Eberwine JE, Abel T, Meaney DF (2014) An open-source toolbox for automated phenotyping of mice in behavioral tasks. Front Behav Neurosci 8:349. 25339878 10.3389/fnbeh.2014.00349 PMC4189437

[B59] Radulescu L, Ferechide D, Popa F (2013) The importance of fetal gender in intrauterine growth restriction. J Med Life 6:38–39. 23599816 PMC3624643

[B60] Robinson EB, Lichtenstein P, Anckarsäter H, Happé F, Ronald A (2013) Examining and interpreting the female protective effect against autistic behavior. Proc Natl Acad Sci U S A 110:5258–5262. 10.1073/pnas.1211070110 23431162 PMC3612665

[B61] Sandin S, Lichtenstein P, Kuja-Halkola R, Larsson H, Hultman CM, Reichenberg A (2014) The familial risk of autism. JAMA 311:1770–1777. 10.1001/jama.2014.4144 24794370 PMC4381277

[B62] Sandman CA, Glynn L, Davis EP (2013) Is there a viability-vulnerability tradeoff? Sex differences in fetal programming. J Psychosom Res 75:327–335. 10.1016/j.jpsychores.2013.07.009 24119938 PMC3796732

[B63] Schaafsma SM, Gagnidze K, Reyes A, Norstedt N, Månsson K, Francis K, Pfaff DW (2017) Sex-specific gene-environment interactions underlying ASD-like behaviors. Proc Natl Acad Sci U S A 114:1383–1388. 10.1073/pnas.1619312114 28115688 PMC5307430

[B64] Shansky RM, Woolley CS (2016) Considering sex as a biological variable will be valuable for neuroscience research. J Neurosci 36:11817–11822. 10.1523/JNEUROSCI.1390-16.2016 27881768 PMC5125240

[B65] Shi L, Fatemi SH, Sidwell RW, Patterson PH (2003) Maternal influenza infection causes marked behavioral and pharmacological changes in the offspring. J Neurosci 23:297–302. 12514227 10.1523/JNEUROSCI.23-01-00297.2003 PMC6742135

[B66] Silverman JL, Babineau BA, Oliver CF, Karras MN, Crawley JN (2013) Influence of stimulant-induced hyperactivity on social approach in the BTBR mouse model of autism. Neuropharmacology 68:210–222. 10.1016/j.neuropharm.2012.07.042 22968082 PMC3522798

[B67] State MW, Levitt P (2011) The conundrums of understanding genetic risks for autism spectrum disorders. Nat Neurosci 14:1499–1506. 10.1038/nn.2924 22037497 PMC3940335

[B68] Straughen JK, Misra DP, Divine G, Shah R, Perez G, VanHorn S, Onbreyt V, Dygulska B, Schmitt R, Lederman S, Narula P, Salafia CM (2017) The association between placental histopathology and autism spectrum disorder. Placenta 57:183–188. 10.1016/j.placenta.2017.07.006 28864010

[B69] Teixeira TP, Queiroga TP, Mesquita MD (2016) Frequency and risk factors for the birth of small-for-gestational-age newborns in a public maternity hospital. Einstein (Sao Paulo) 14:317–323. 10.1590/S1679-45082016AO3684 27759818 PMC5234741

[B70] Thomas A, Burant A, Bui N, Graham D, Yuva-Paylor LA, Paylor R (2009) Marble burying reflects a repetitive and perseverative behavior more than novelty-induced anxiety. Psychopharmacology 204:361–373. 10.1007/s00213-009-1466-y 19189082 PMC2899706

[B71] Treit D, Fundytus M (1988) Thigmotaxis as a test for anxiolytic activity in rats. Pharmacol Biochem Behav 31:959–962. 10.1016/0091-3057(88)90413-3 3252289

[B72] Walker CK, Krakowiak P, Baker A, Hansen RL, Ozonoff S, Hertz-Picciotto I (2015) Preeclampsia, placental insufficiency, and autism spectrum disorder or developmental delay. JAMA Pediatr 169:154–162. 10.1001/jamapediatrics.2014.2645 25485869 PMC4416484

[B73] Wegiel J, Kaczmarski W, Flory M, Martinez-Cerdeno V, Wisniewski T, Nowicki K, Kuchna I, Wegiel J (2018) Deficit of corpus callosum axons, reduced axon diameter and decreased area are markers of abnormal development of interhemispheric connections in autistic subjects. Acta Neuropathol Commun 6:143. 10.1186/s40478-018-0645-7 30567587 PMC6299595

[B74] Werling DW, Geschwind DH (2013) Sex differences in autism spectrum disorders. Curr Opin Neurol 26:146–153. 10.1097/WCO.0b013e32835ee548 23406909 PMC4164392

[B75] Yang M, Crawley JN (2009) Simple behavioral assessment of mouse olfaction. Curr Protoc Neurosci Chapter 8:Unit 8.24. 10.1002/0471142301.ns0824s48 PMC2753229 19575474

[B76] Zerbo O, Qian Y, Yoshida C, Grether JK, Van de Water J, Croen LA (2015) Maternal infection during pregnancy and autism spectrum disorders. J Autism Dev Disord 45:4015–4025. 10.1007/s10803-013-2016-3 24366406 PMC4108569

