# Supplementary figures and images for: “Females Are Not Just ‘Protected’ Males”: Sex-Specific Vulnerabilities in Placenta and Brain after Prenatal Immune Disruption
Source: eNeuro. 2019 Nov 6;6(6):ENEURO.0358-19.2019. doi: 10.1523/ENEURO.0358-19.2019 (PMC6838689; doi:10.1523/ENEURO.0358-19.2019)

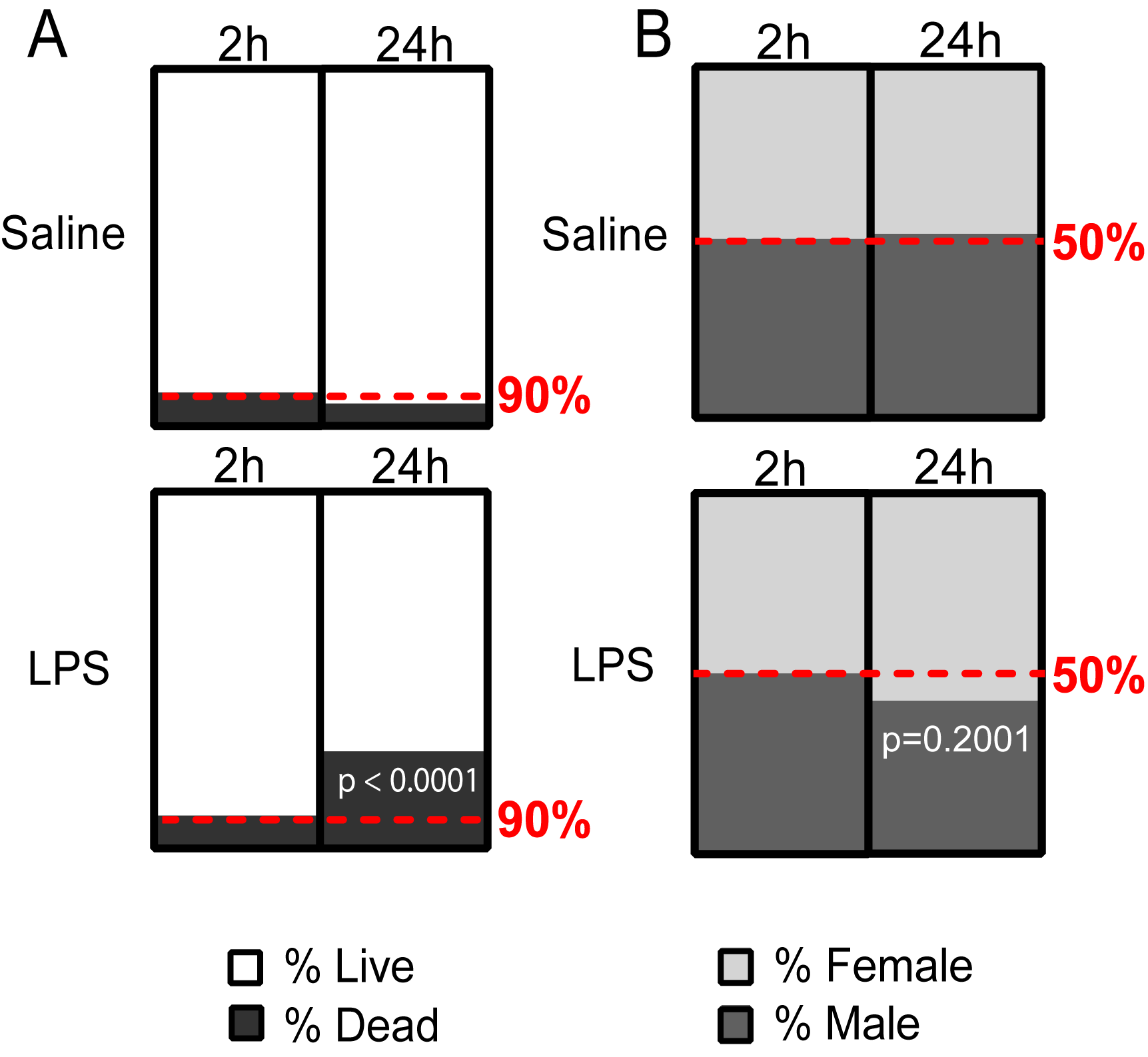

Supplement: Figure 1-1 — Fetal loss and sex ratios 24 h after LPS. A, Proportion of live and dead fetuses 24 h after saline or LPS 60 μg/kg treatment were determined by gross examination. Blanched and resorbing fetuses were counted as dead, while pink and intact fetuses were considered alive at the time of dissection. B, Sex ratio of surviving fetuses was determined by PCR genotyping of Jarid1c/d in fetal tissue (p values from Fisher’s exact test). Download Figure 1-1, TIF file. [file sup_enu-eN-NWR-0358-19-s01.tif]

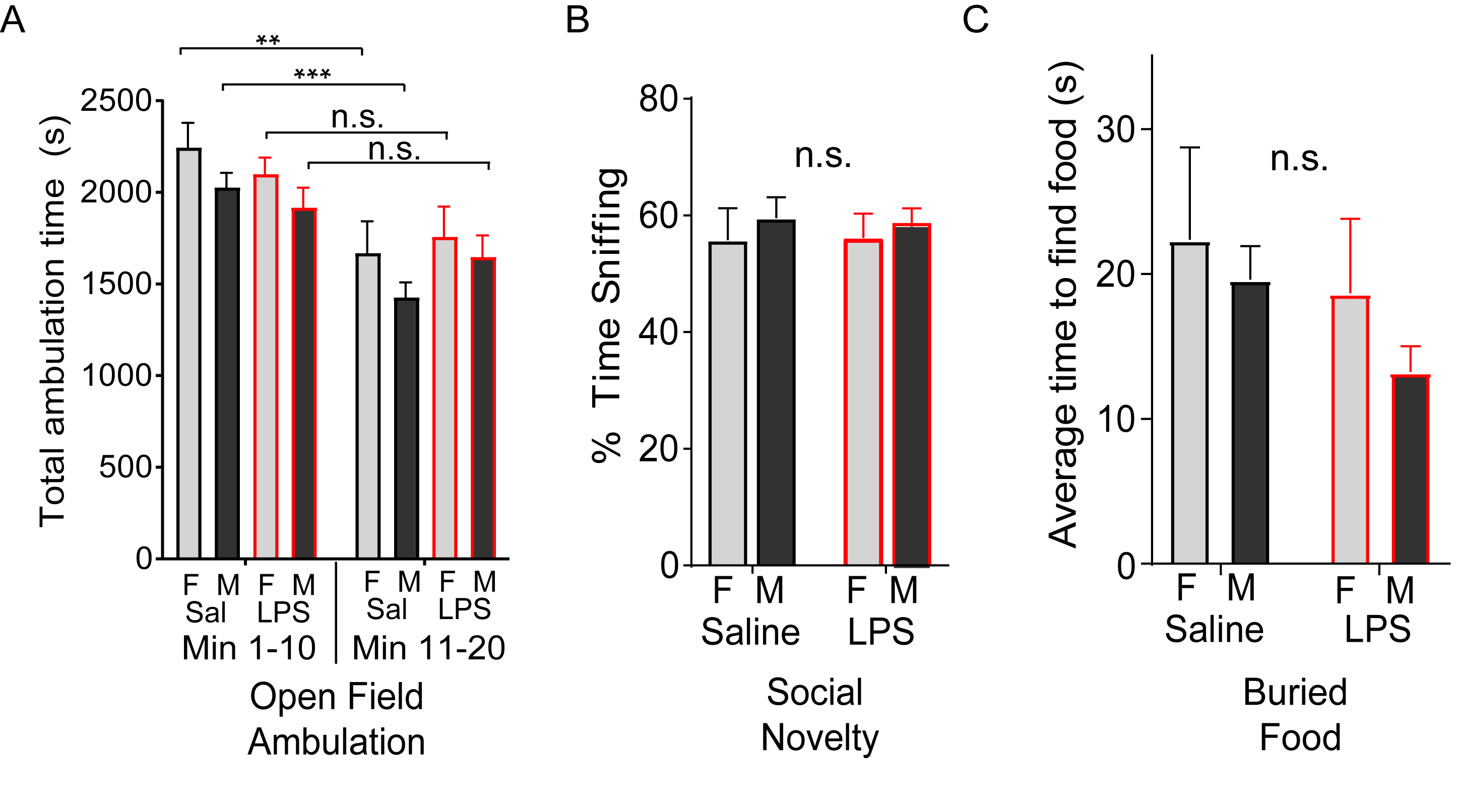

Supplement: Figure 4-1 — Activity, social novelty, and olfaction. Some behavioral tests revealed subtle or no sex differences. A, On an open field task of exploration and anxiety, mice were allowed to explore an open chamber with an unsheltered central area. Total ambulation time over the course of the trial, presented as early and late 10 min phases. B, Subsequent to the three-chamber social interaction test, mice were exposed to a novel mouse under the previously empty cup, and preference is reported as the percentage of time spent interacting with the novel mouse over the familiar mouse, an indicator of social memory. C, The open field cohort underwent a buried food test, and the time to find cereal buried in cage bedding was recorded. Comparisons were made by two-way ANOVA for social memory and buried food, and repeated-measures ANOVA with post hoc for ambulation: **p < 0.01, ***p < 0.001, n.s. = not significant. Download Figure 4-1, TIF file. [file sup_enu-eN-NWR-0358-19-s02.tif]
